# Resistance of a short-term memory concealed information test with famous faces to countermeasures

**DOI:** 10.3758/s13421-023-01489-1

**Published:** 2023-12-05

**Authors:** Hugues Delmas, Camélia Ciocan, Mariya Novopashyna, Céline Paeye

**Affiliations:** 1 Université Sorbonne Paris Nord, 93430 Villetaneuse, Paris, France; 2 Université Paris Cité, Vision Action Cognition, Boulogne-Billancourt, Paris, France

**Keywords:** Concealed information test, Face processing, Eye movements, Classification analyses

## Abstract

The concealed information test (CIT) aims at identifying knowledge that a person wants to hide, by measuring physiological indices during the presentation of known versus unknown items. Recently, Lancry-Dayan et al. (*Journal of Applied Research in Memory and Cognition*, 7 (2), 291–302, 2018) proposed a new version of this test that included a short-term memory task to maximize differences between responses to items. Participants were asked to memorize four pictures of faces that included one face of an acquaintance. The authors observed that participants looked at the familiar face during the first second and then tended to avoid it. This specific orientation-avoidance pattern occurred even in participants instructed to conceal their familiarity with the known faces (in a spontaneous or a guided manner). In a first experiment, we reproduced Lancry-Dayan et al.’s (2018) study using photos of famous faces. The pattern found by Lancry-Dayan et al. was observed in participants asked to perform the memory task only, participants asked to conceal their familiarity with the famous faces, and participants of a countermeasure group. In a second experiment, we tested the robustness of Lancry-Dayan et al.'s countermeasure. We modified the instructions by emphasizing the oculomotor task or giving feedback. While between-group differences in gaze-pattern appeared after feedback was provided, classification analyses were still able to distinguish between familiar and unfamiliar faces accurately, which revealed the good resistance of this new CIT protocol to countermeasures.

**Supplementary Information:**

The online version contains supplementary material available at 10.3758/s13421-023-01489-1.

## Introduction

Approximately 5,000 Concealed Information Tests (CITs) are conducted each year by the Japanese police force to assist investigators in the search for truth (Ben-Shakhar, 2012). The CIT was designed to detect if a person is concealing knowledge about the relevance of an item (Ben-Shakhar, 2012). Classically, familiar and unknown items are presented sequentially to observers while various physiological or behavioral indices are measured. These indices can include measures of the autonomic nervous system (Gamer, 2011), brain evoked potentials (Rosenfeld, 2019), or reaction times (Varga et al., 2014). These responses, known to be difficult to control, change when a familiar item is presented (Matsuda et al., 2012). Variations are larger in people who know the critical item (which is of course not sufficient to establish guilt).

Recently, eye tracking was introduced in the protocol, affording the opportunity for new measurements. In classic CITs, ocular fixations are recorded during the sequential presentation of familiar and unfamiliar faces, while observers indicate whether they know the faces (Millen et al., 2017). Eye movements can also be recorded during the parallel presentation of several faces (Schwedes & Wentura, 2012). In such CITs, fewer and longer fixations were observed on familiar than on unfamiliar faces, even when participants were asked to conceal their familiarity.

To increase the differences between eye movements made towards familiar versus unfamiliar faces, Lancry-Dayan et al. (2018) added a short-term memory task to the CIT protocol. We will call this CIT version the *short-term memory CIT* (STM-CIT) in contrast to the classic CIT described above. Participants were asked to memorize four pictures of faces displayed simultaneously for 5 s. In 50% of the trials, the display included the face of an acquaintance. The display was then replaced by a single face (acquaintance or not). Participants had to indicate whether this single face was present in the preceding four-face display.

Lancry-Dayan et al. (2018) observed that during the four-face displays, participants oriented their gaze towards the familiar face during the first second and then tended to avoid it. This avoidance effect was probably due to observers' attentional focus on unfamiliar faces, in their effort to memorize them (Jackson & Raymond, 2008). This orientation-avoidance pattern was observed when participants were instructed to memorize the faces only (control experiment), when they were asked to conceal their familiarity with the acquaintances’ faces (concealed experiment), and even when they were advised to look equally at all faces in order to thwart the test (countermeasure experiment). Furthermore, Support-Vector Machine (SVM) classifiers categorized familiar and unfamiliar faces with accuracies above 88%, regardless of the instructions given to the participants. In other words, the STM-CIT appeared to be resistant to countermeasures, which was encouraging for concealed information detection.

The degree of familiarity is known to modulate eye movements during a CIT (Lancry-Dayan et al., 2021; Millen et al., 2017). For instance, in a classic CIT, Millen et al. (2017) observed fewer fixations for both personally familiar or celebrity faces than for unknown faces. Recently, in another STM-CIT study, Lancry-Dayan et al. (2021, Experiment 2) used newly learned objects in addition to personally significant objects (i.e., objects owned by the participants) as familiar items. The preference effect towards familiar stimuli was observed only for personally significant objects, likely because these objects carry more motivational or emotional values. It is also possible that other factors, such as richer contextual information or the number of previous exposures, facilitate a deeper encoding in long-term memory.

The goal of our first experiment was to test whether the aforementioned STM-CIT studies could be reproduced with another kind of familiar item: faces of celebrities, rather than faces of acquaintances (Lancry-Dayan et al., 2018), or objects (Lancry-Dayan et al., 2021). If this is the case, we should observe an orientation-avoidance ocular pattern during the four-face displays (i.e., observers would first orient their gaze towards famous faces, and then tend to avoid them). In addition, ocular fixations on famous faces during the single-face displays should be longer than fixations on non-famous faces, consistent with previous studies showing that familiarity with an item extends fixation duration (Althoff & Cohen, 1999; Heisz & Shore, 2008; Ryan et al., 2007; Schwedes & Wentura, 2012). Finally, we expected classification algorithms to distinguish between celebrities and unknown faces with high efficiency, in line with Lancry-Dayan et al. (2018, 2021).

The lack of an instruction effect in Lancry-Dayan et al.’s (2018) study is puzzling as countermeasures are known to alter eye movement patterns in classic CIT (Peth et al., 2016). Interestingly, in their 2021 study (Experiment 3), Lancry-Dayan and her colleagues instructed participants to conceal their familiarity with the personally known items by serially scanning the images, in addition to looking equally at each object. They observed that gaze behavior was only partially modified by these instructions. The goal of Experiment 2 was to test the robustness of Lancry-Dayan et al.’ (2018, 2021) countermeasures by proposing in addition to instructions some explanations on the expected patterns of eye movements or feedback on participants’ oculomotor performance. Such detailed instructions should lead participants to modify their eye movements (Maes, 2003; Souza et al., 2012). In particular, they should be able to reduce the avoidance effect (assumed to be less controllable than the orienting response) during the four-face displays.

## Methods

The general design of our experiments was similar to that of Lancry-Dayan et al. (2018), except that we used pictures of celebrities instead of acquaintances. Participants were first asked to memorize four faces displayed simultaneously (one of these faces was a celebrity in 50% of the trials). These faces were then replaced by a single face, famous or not. Participants had then to indicate whether this face was present in the preceding display. Groups of participants differed according to the instructions they received.

The first experiment involved three groups of participants. In the *Control* group, the participants were instructed to perform the memory task only. In the *Concealment* group, they received the same instructions and were also asked to conceal their familiarity with the celebrity faces. No further guidance was provided. The *Simple countermeasure* group received instructions identical to those given to the Concealment group, with the addition of concise indications on how to conceal familiarity: participants were advised to direct their gaze equally to all faces. In the second experiment, the instructions given to the other two countermeasure groups were more precise. Firstly, they emphasized the importance of both the memory and the eye-movement tasks: participants were shown a slideshow presenting the orientation-avoidance ocular pattern observed by Lancry-Dayan et al. (2018). Furthermore, in the middle of the experimental session, participants in the *Enhanced countermeasure* group were presented with these results again, whereas participants of the *Feedback* group were shown a graph presenting the time course of their own gaze position averaged over their first 32 trials.

### Participants

Participants were recruited through the online platform of the Institute of Psychology. They were students of this Institute, all naive about the purpose of the study, and obtained course credits for their participation (no other incentive was provided, even in the Concealment or countermeasure groups). They had normal or corrected-to-normal vision, and were excluded from the study if they took any medication affecting memorization or vigilance. They gave their informed written consent prior to the experiment. Experiments were in accordance with the principles of the Declaration of Helsinki. Approval was granted by the Ethics Committee of University Paris Cité (2022-4-PAEYE; IRB 00012022-4).

The study was registered before data analyses in the Open Science Framework (OSF) registry (registration DOI: 10.17605/OSF.IO/85ZMY) available via the following link: https://osf.io/85zmy.

We recorded the data of 15 participants per group.^1^ A subsequent sensitivity power analysis, performed with the software program G*power (Faul et al., 2007), revealed that a 2 × 3 ANOVA with 15 participants per group (45 participants in total, for whom we obtained one preference index per trial phase, see below) was sensitive to detect effects of η^2^_p_ = .20 with 80% power (alpha = .05).

Forty-nine participants (ten males) aged 18–40 years volunteered to perform the first experiment. Four participants were excluded because of failure to understand instructions (1) or because of calibration failure (3). The average age of the 45 remaining participants was 22.5 years (*SD* = 4.9). Thirty-two participants (four males) aged 18–40 years volunteered to perform the second experiment. Two participants were excluded because the data files were damaged or because of calibration failure. The average age of the 30 remaining participants was 19.8 years (*SD* = 4.2). Table 1 presents details of the 15 participants in each group.

**Table 1:** Details of participants in each group

Group	Control	Concealed	Simple countermeasure	Enhanced countermeasure	Feedback
Number of males	5	2	3	1	2
Mean ages (± *SD*s)	23.9 (± 2.6)	21.7 (± 5.5)	22.7 (± 5)	19.1 (± 1.6)	20.5 (± 5.8)

### Stimuli and material

Participants were seated in a dimly lit room, 57 cm from a screen, with their head stabilized by a chin and forehead rest. The stimuli, generated with the Psychophysics Toolbox for Matlab (Brainard, 1997; Kleiner et al., 2007; Pelli, 1997), were displayed on a 24.5-in. BENQ screen (ZOWIE XL-2540), with a refresh rate of 144 Hz and a spatial resolution of 1,920 × 1,080. They were displayed on a gray screen (luminance 31 cd/m^2^). Eye movements were monocularly recorded with an EyeLink^®^ 1000 (SR Research^®^, Ontario, Canada) sampling at 1,000 Hz. A black cross (1° × 1°, line width 3 pixels) was used as a fixation point to ensure that participants started the memorization and recognition tasks by looking at the same location on the screen.

Among the 64 pictures presented during the experiments, 56 were pictures of unknown faces (28 men and 28 women) retrieved from two public databases (Ebner et al., 2010; Vieira et al., 2014). The eight remaining pictures were faces of four men (François Hollande, Emmanuel Macron, Nicolas Sarkozy, and Donald Trump) and four women (Catherine Deneuve, Brigitte Macron, Angela Merkel, and Vanessa Paradis) who were famous in France. Prior to the experiment, these celebrities were recognized correctly by 100% of 23 persons aged between 18 and 40 years (who did not participate in the present study).

All images were vertical 11.5° × 8.6° pictures of full-frontal faces taken from the same distance (the hair had to be cut from some pictures, so that the eyes, nose, and mouth occupied the same location on all pictures), with a neutral expression and no jewels (necklaces and earrings were cropped). The celebrities’ faces were not heavily made-up. The picture background was the same uniform gray as the screen background. The images can be found on the OSF at https://osf.io/vygfz/.

### General design and procedure

The experiment, which lasted about 45 min, began with four practice trials followed by 64 STM-CIT trials. After the test, participants answered questions about the pictures (the experimental conditions are further detailed below). When eye movements were recorded, subjects performed first a calibration procedure: they were required to track a point successively presented at nine different locations around the screen. During trials, fixation was checked automatically before the presentation of each display. Gaze had to be inside an invisible window of 2° × 2° centered on the fixation cross for a display to appear (a saccade onset triggered the instruction to fixate the cross and reset the checking period), so that participants began each task by looking at the same screen location.

**Fig. 1:**
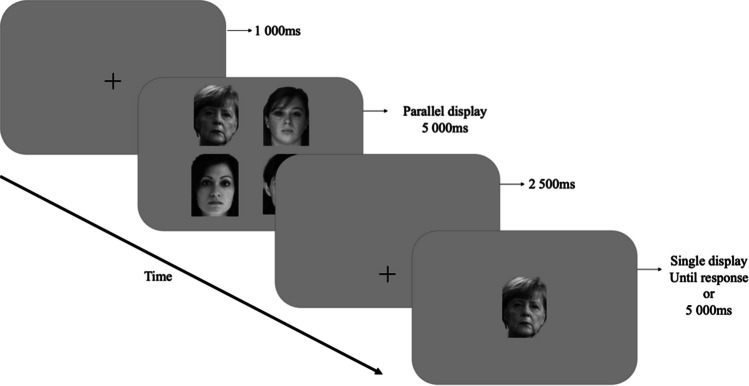
Structure of trials. After a central fixation, a *parallel display* of 2 × 2 faces, equally distributed over the screen, was presented. A new fixation cross then appeared 11.4° below the center of the screen. Fixation was then checked again and a *single-face display* appeared at the center of the screen. This picture remained on the screen (for a maximum of 5,000 ms) until participants indicated whether they had seen the face in the parallel display or not

Each practice and STM-CIT trial started with the presentation of a central fixation cross (Fig. 1, frame 1). After 1,000 ms, during which central fixation was checked, a *parallel display* of 2 × 2 faces, equally distributed over the screen, was presented for 5,000 ms (frame 2). The centers of these pictures were located at 18° eccentricity from the center of the screen. A new fixation cross then appeared 11.4° below the center of the screen (frame 3). Participants had 2,500 ms to displace their gaze towards this cross. Fixation was then checked again for 500 ms and a *single-face display* (a face of the same size as the pictures presented in the first parallel display) appeared at the center of the screen (frame 4). This picture remained on the screen until participants indicated whether they had seen the face in the previous parallel display or not. They did so by pressing respectively the “O” or “N” keys of a regular keyboard. If participants did not answer within 5,000 ms, the trial was considered as a “no response” (on average, 0.46% of the trials, *SD* = 1.17%). Data from these trials were considered in eye-movement analyses of the parallel displays but were removed from the analyses of ocular and manual responses to the single-face display. A new central fixation cross signaled the beginning of the next trial.

The practice trials were the same for all participants. They were designed to familiarize them with the procedure and to train them in the short-term memory task. The faces used in these four trials were not presented later in the test (pictures of Arnold Schwarzenegger and Julia Roberts were used as famous faces).

The test consisted of six different types of trials, depending on the presence versus absence of a famous face in the parallel and single-face displays (see Table 2). Combinations of faces and their respective location on the parallel displays were pseudorandomized, with the following constraints. Firstly, faces of the same gender were presented in a trial. Secondly, each face appeared three or four times in the parallel displays during the experiment (each famous face appeared four times, each time in a different location). In addition, each famous face appeared four times in the single-face displays, whereas non-famous faces appeared only once. Finally, the correct answer was “yes” in half of the trials. The order of trials was randomized.

**Table 2:** Types of trials in both experiments

Trial type	1	2	3	4	5	6
Famous face in the parallel display	yes	yes	yes	no	no	no
Famous face in the single-face display	yes	no	no	yes	no	no
Correct answer	yes	yes	no	no	yes	no
Number of trials	16	8	8	16	8	8

The experiments ended with the sequential presentation of each picture. Eye movements were not recorded. Participants had to indicate whether they considered that the face was famous by pressing the “O” (if the face was famous) or “N” keys of the keyboard.

### Eye-movement and data analysis

For eye-movement analyses, we used the Eyelink parser to identify the onset and offset of saccades, using 30°/s velocity and 8,000°/s^2^ acceleration thresholds. Samples identified as blinks were removed from the eye-movement traces. Samples gathered from time intervals between saccades and blinks were defined as fixations. Saccades and fixations outside a picture (i.e., landing in the uniform gray background) were not considered in the analyses.

A non-famous face that was erroneously identified as a celebrity face by a participant during the post-experiment session (i.e., a false recognition) was considered in our analyses as being familiar to this participant, whereas a famous face that was not identified (i.e., a miss) was considered as a non-familiar face. The number of participants who made at least one such identification error and the proportions of trials in which the parallel display contained a misidentified face are reported in Table 3. Trials with more than one false recognition in the parallel display, or with one celebrity face and one false recognition, were discarded from the analyses (overall, 0.44% of trials, *SD* = 1.64%).

**Table 3:** Mean proportions (± *SD*s) of the 64 STM-CIT trials in which a false recognition (i.e., when a non-famous face was erroneously identified as a celebrity face) or a miss (i.e., when a famous face was not identified) occurred, in each group of participants

Group of participants	Control	Concealment	Simple counter- measure	Enhanced counter- measure	Feedback
False recognition trials	6.98% (± 15.18) *n* = 5	5.63% (± 10.31) *n* = 6	9.38% (± 17.83) *n* = 5	3.33% (± 4.53) *n* = 6	2.19% (± 3.38) *n* = 6
Miss trials	0.42% (± 1.61) *n* = 1	1.25% (± 3.51) *n* = 2	1.25% (± 3.51) *n* = 2	0.21% (± 0.81) *n* = 1	0.94% (± 2.27) *n* = 3

The numbers *n* indicate the number of participants who made at least one error

It is possible that false recognitions occurred because participants were presented with unknown faces several times during the STM-CIT. Therefore, it is not known whether falsely recognized faces were actually mistaken for celebrity faces, or whether they were encoded in long-term memory over the test, thereby acquiring the status of “newly learned” (Lancry-Dayan et al., 2021) faces. To confirm that our results are robust and that they are not driven by these misidentification trials, we performed the same analyses, but after removing the trials in which a misidentified face had been presented. These analyses are presented in the Online Supplementary Material (OSM).

#### Eye movement analyses during the four-face parallel displays

Only parallel displays containing a famous face were considered in these analyses. To visualize gaze allocation during these displays, we divided the 5,000-ms display duration into 50 100-ms bins and computed the proportion of time that gaze was directed to famous versus unknown pictures in each bin, as in Lancry-Dayan et al.'s (2018) study. Following these authors, we defined two phases: 200–1,000 ms (first phase) and 1,001–5,000 ms (second phase). For each trial and each participant, we extracted the number of fixations and the dwell time (i.e., the time spent) on each face, and for each phase. The number of fixations and dwell time on the three non-famous faces were pooled and divided by three to make them comparable to the number of fixations and dwell time on the famous faces. We computed a *preference index*, corresponding to the signed difference between the average proportions of time spent on famous faces and of time spent on unknown faces. A positive (negative) value indicated that participants fixated longer on famous (unknown) faces.

Mixed analyses of variance (ANOVAs) were run on preference indices and differences between the mean number of fixations on famous versus unknown faces, with one between-subjects factor (the factor Group, detailed below) and two within-subjects factors: Phase (first vs. second) and Faces (famous vs. unknown). Where appropriate, between- and within-group comparisons were run using Tukey post hoc tests (with *p-*values adjusted for families of 15 estimates). To identify orientation responses and avoidance effects, we performed one-sample *t*-tests (or Wilcoxon signed-rank tests when the normality assumption was not met) comparing the preference indices of each phase against the zero value. A type I error rate of 0.05 was adopted for these analyses. In order to corroborate the absence of effects we also conducted Bayesian repeated-measures ANOVAs (JASP Team, 2022, v. 0.16.4.0). In these analyses, we compared the null model that contains only the grand mean to each of the models that could be created by including or not a main effect (Group and Phase) or their interaction. Results are expressed as Bayes factors (*BF*_10_) for each model against the null model. Following Van den Bergh et al. (2022), repeated-measures ANOVA models included random slopes, and we used a uniform prior (Rouder et al., 2012) whose values were set to the JASP default values (*r* scale fixed effects = 0.5; *r* scale random effects = 1; *r* scale covariate = 0.354, corresponding respectively to the values of the hyperparameter *r*, specified separately for the groups of fixed effects, random effects, and covariates). To quantify the absence of evidence for the Group × Phase interaction, we compared the model containing the interaction to the model with the two predictors – stripped of the interaction, as recommended by Mathôt (2017) and van den Bergh et al. (2020) – which yielded *BF*_incl_ scores.

#### Analyses of ocular and manual responses during the single-face displays

All trials (except when no response was provided in the requested time-window) were considered to measure mean fixation duration, mean reaction time, and mean proportion of correct responses for each participant and each face (famous vs. unknown).

We conducted mixed ANOVAs with one between-subjects factor (Group) and one within-subjects factor (Face: famous vs. unknown), as well as Bayesian repeated-measures ANOVAs (see above) to quantify evidence in favor of our null hypotheses.

#### Classification analyses

Our classification analyses followed the analysis performed by Lancry-Dayan et al. (2018), using a SVM classifier. The main purpose of the CIT is to differentiate between knowledgeable and unknowledgeable individuals (guilty and innocent in its forensic application). This differentiation is based on within-individual differences between the relevant (in this case, famous) and control (unknown) items. Therefore, the target variable of our classification analyses was the Type of faces. Because there were no unknowledgeable participants in this study, we simulated such a sample by extracting trials in which a famous face (or a misidentified face) did not appear (Lancry-Dayan et al., 2018, 2021; see also Meijer et al., 2007). Therefore, we compared trials containing a celebrity face to trials in which no celebrity face appeared.

We used the following predictors: dwell time measured over (a) the first phase and (b) the second phase, (c) total fixation count, and (d) total number of visits during the parallel displays. A visit was defined as consecutive fixations on a specific picture before a saccade was made outside of that picture.

One potential bias that may arise in classification analyses is overfitting (Kleinberg et al., 2019), which occurs when the model detects a pattern within the data provided but cannot generalize it to unseen data. To minimize this risk, we divided our dataset into two separate training and test sets. Predictor variables were standardized on the training set. Then, the mean and standard deviation of the training set were used to standardize the predictors of the test set (Müller & Guido, 2016). Classification analyses were performed using the *ScikitLearn* Python library (Pedregosa et al., 2011). For the training set, we used the *GridSearch* method, with a radial-based function (RBF) as SVM kernel and a 4 K-folds for cross-validation (whose purpose was also to avoid overfitting; Kleinberg et al., 2019). That is, the dataset was split into four folds of 16 images, with three folds used for training and one for testing. Each fold was used successively for testing during the cross-validation. We tested the hyperparameters C: [0.001, 0.01, 0.1, 1, 10, 100, 1000], and gamma: [0.001, 0.01, 0.1, 1, 10, 100, 1000]. Then we selected the best model based on the training set and applied it to the test set to get the areas under the receiver operating characteristic curves (ROC AUCs). ROC AUC is a statistically consistent and discriminating measure for evaluating classifier performance (Fawcett, 2006; Hossin & Sulaiman, 2015; Ling et al., 2003). This measure, which assesses the classification model's ability to discriminate between the two types of faces (famous and unknown), is generated by plotting sensitivity (the number of true positives divided by the sum of true positives and false negatives) against the false-positive rate (the number of false positives divided by the sum of false positives and true negatives). A value of 1 indicates the best discrimination, whereas a value of 0.5 indicates random discrimination.

For descriptive purposes, we calculated balanced accuracies and F1 scores, which are also often used in machine learning to evaluate classifier performance. Balanced accuracy was computed for each participant by averaging sensitivity and specificity (the number of true negatives divided by the sum of false positives and true negatives). Compared to accuracy, this measure reduces the risk of overestimating classifier performance when a dataset is imbalanced (Brodersen et al., 2010). The F1 score is the harmonic mean of the specificity and the precision (the number of true positives divided by the sum of true positives and false positives). Both measures range from 0, indicating the worst performance, to 1, indicating the best performance. ROC AUCs, balanced accuracies, and F1 scores were obtained using the following *Scikitlearn* library commands: *sklearn.metrics.balanced_accuracy_score*, *sklearn.metrics.roc_auc_score* and *sklearn.metrics.f1_score*, respectively.

The code of these analyses as well as the source files are available via the OSF at: https://osf.io/vygfz/.

The construction of the training and test sets depended on the type of analyses conducted: within or between participants. Within-participants analyses use a subset of a participants’ data to predict their familiarity with specific faces. We trained the SVM GridSearch on one participant and obtained ROC AUCs, balanced accuracies, and F1 scores. This operation was repeated for each participant. In contrast, between-participants analyses predict a participant’s familiarity with specific faces based on the data of other participants. We trained the SVM GridSearch on all participants minus one (N − 1) and applied the best model to the remaining participants to obtain ROC AUCs, balanced accuracies, and F1 scores. This operation was repeated for each participant.

As in Lancry-Dayan et al. (2018), we compared (using two-sample *t-tests*) the three measures of classification performance to baseline values, obtained as follows. For each group of participants, we ran the classifier used in the main analyses on a set of images containing the 64 faces randomly tagged as famous or unknown, and distributed according to the original proportion of famous and unknown faces (8/64 and 56/64, respectively). Table 4 presents the ROC AUCs, balanced accuracies, and F1 scores obtained from this random distribution. Finally, to test the effect of instructions on classification efficiency, one-way ANOVAs were run on the classification indices, with Group as a between-subjects factor (unregistered analyses).

**Table 4:** Baseline values (means and standard deviations) of experiments obtained from the random distribution, for each group and each type of classification analysis

	Within participants			Between participants		
*Experiment 1*						
	Control	Concealment	Simple C.	Control	Concealment	Simple C.
ROC AUC	.38 (0.22)	.45 (0.21)	.58 (0.23)	.60 (0.14)	.64 (0.15)	.73 (0.10)
Balanced accuracy	.50 (0)	.49 (0.02)	.50 (0.02)	.51 (0.02)	.51 (0.02)	.53 (0.04)
F1 score	0 (0)	0 (0)	0 (0)	.03 (0.07)	.03 (0.07)	.11 (0.14)
*Experiment 2*						
	Simple C.	Enhanced C.	Feedback	Simple C.	Enhanced C.	Feedback
ROC AUC	.57 (0.29)	.39 (0.22)	.43 (0.28)	.56 (0.11)	.61 (0.11)	.82 (0.08)
Balanced accuracy	.49 (0.01)	.48 (0.04)	.49 (0.03)	.50 (0.02)	.50 (0.01)	.59 (0.08)
F1 score	0 (0)	0 (0)	0 (0)	.01 (0.06)	0 (0)	0.26 (0.18)

Simple C. = Simple countermeasure; Enhanced C. = Enhanced countermeasure

## Experiment 1

In this first experiment we tested whether the orientation-avoidance ocular pattern reported by Lancry-Dayan et al. (2018) would be observed with celebrity faces.

As mentioned above, 45 participants were assigned to three different groups that were given the same instructions as in Lancry-Dayan et al.’s study (2018). In the *Control* group, participants were instructed to perform the memory task only. In the *Concealment* group, participants were also asked to conceal their familiarity with the celebrity faces, without receiving any explanation as to how to proceed. In the *Simple countermeasure* group, participants received both instructions and were also advised to direct their gaze equally to all faces.

### Results

#### Eye movements during the four-face parallel displays

**Fig. 2:**
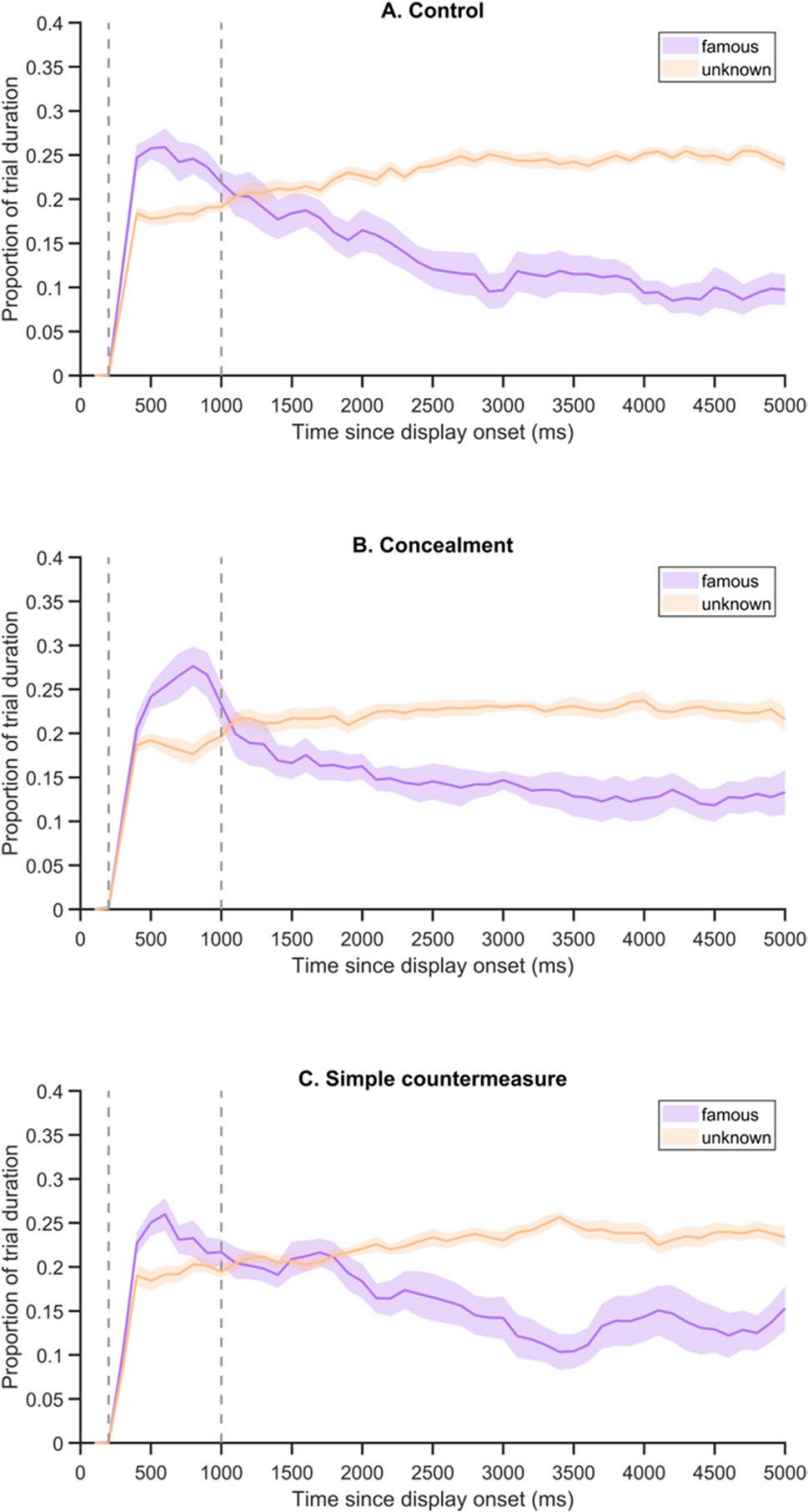
Time course of gaze position for (**A**) the Control, (**B**) the Concealment, and (**C**) the Simple countermeasure groups of Experiment 1. Proportion of time spent on famous vs. unknown faces during the first phase (200–1,000 ms) and second phase (1,001–5,000 ms) of the four-face parallel displays. Time spent on the unknown faces of a trial was averaged across the three pictures. Shadowed areas indicate ± SEM across participants, and dashed lines the beginning of each trial phase (color figure online)

Overall, the time course analyses (Fig. 2) showed that during the first trial phase, gaze was initially attracted by famous faces, whereas in the second phase (beginning after 1,000 ms), participants spent less time on these faces. These gaze patterns were similar in each group.

**Fig. 3:**
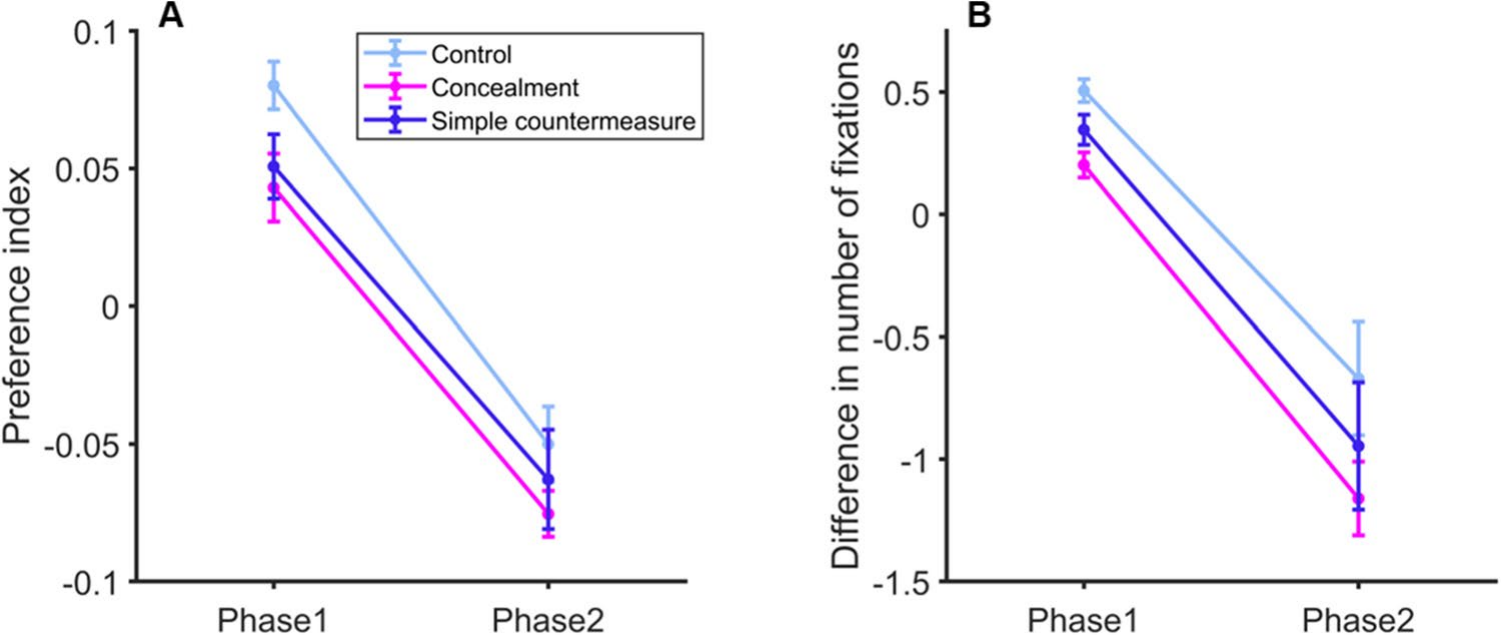
(**A**) Mean preference indices and (**B**) differences between the mean number of fixations on famous vs. unknown faces, obtained for each group in Experiment 1, in each phase of the four-face parallel displays. Error bars: SEM (color figure online)

A 2 × 3 ANOVA on preference indices with the within-participants factor Phase and the between-participants factor Group confirmed the significant effect of Phase*, F*(1,42) = 173.54, *p* < .001, η^2^_p_ = 0.81. Orientation and avoidance effects are shown in Fig. 3. Mean preference indices of the first phase (*M*_*Index*_ = 0.06; *SD =* 0.05) were greater than zero for the three groups, all *p*s < .01, while those of the second phase (*M*_*Index*_ = -0.06; *SD =* 0.05) were all smaller than zero, all *p*s < .01 (Fig. 3A). We found moderate evidence for the lack of a Group effect, *F*(2,42) = 2.70, *p* = .079, *BF*_10_ = 0.27, as well as for the absence of interaction, *F*(2,42) = .29, *p* = .75, *BF*_incl_ = 0.21.

Similar statistical conclusions can be drawn from the analysis of differences between the mean number of fixations on famous versus unknown faces. The effect of Phase was significant, *F*(1,42) = 102.24, *p* < .001, η^2^_p_ = 0.71 (Fig. 3B). In the first phase, these differences (*M*_*deltaFix*_ = 0.35; *SD =* 0.24) were greater than zero for the three groups, all *p*s < .001, while they were all smaller than zero in the second phase (*M*_*deltaFix*_ = -0.97; *SD =* 0.24, all *p*s < .01). No effect of Group, *F*(2,42) = 1.35, *p* = .271, *BF*_10_ = 0.19, and no interaction, *F*(2,42) = 0.03, *p* = .98, *BF*_incl_ = 0.15, were observed (evidence in favor of these null hypotheses is, however, moderate).

Note that when statistical analyses excluded trials in which a misidentified face had been presented (i.e., when data include only trials in which the faces’ status was totally unambiguous), the conclusions did not change, except that the level of evidence for the absence of the two aforementioned interactions decreased and became anecdotal (see OSM, Section 1.1).

#### Ocular and manual responses in the single-face displays

**Fig. 4:**
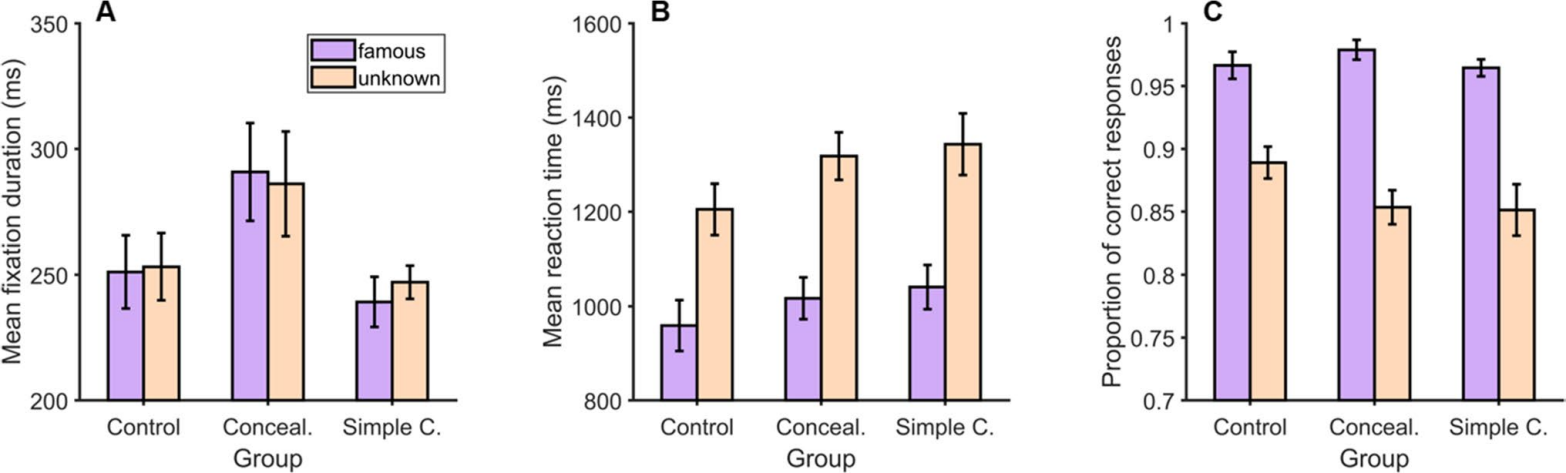
(**A**) Mean fixation durations, (**B**) mean reaction times, and (**C**) mean proportions of correct responses in the short-term memory task for famous and unknown faces, and for each group of participants. Error bars: SEM. *Conceal*.: Concealment group; *Simple C*.: Simple countermeasure group (color figure online)

To examine whether eye movements differed during the exploration of famous versus unknown faces, we compared the mean durations of fixations made on each type of face, for each group of participants (Fig. 4A). Contrary to our hypotheses, the factor Face had no effect on fixation durations, *F*(1,42) = 0.197, *p* = .66, *BF*_10_ = 0.23. Overall, fixations on famous and unknown faces lasted 260.4 ms (*SD* = 61.5 ms) and 262.1 ms (*SD* = 58.6 ms), respectively. Our data revealed inconclusive evidence regarding the absence of a main effect of Group, *F*(2,42) = 2.71, *p* = .078, *BF*_10_ = 0.96, or an interaction between the two factors, *F*(2,42) = 0.88, *p* = .42, *BF*_incl_ = 0.43.

As hypothesized, participants responded faster (Fig. 4B), *F*(1,42) = 144.84, *p* < .001, η^2^_p_ = 0.78, and more accurately (Figure 4C), *F*(1,42) = 104.52, *p* < .001, η^2^_p_ = 0.71, when a famous face (*M*_*ReactionTime*_ *=* 1,005.5 ms, *SD =* 187.8 ms, *M*_*Proportion_correct*_ *=* 0.97, *SD =* 0.03) than when an unknown face (*M*_*ReactionTime*_ *=* 1,288.7 ms, *SD =* 225 ms, *M*_*Proportion_correct*_ *=* 0.87, *SD =* 0.06) was presented in the single-face display. The factor Group did not affect reaction times, *F*(2,42) = 1.38, *p* = .26, *BF*_10_ = 0.35, nor the proportions of correct responses, *F*(2,42) = 1.17, *p* = .31, *BF*_10_ = 0.18. Interactions between the two factors were not significant, neither for reaction times, *F*(2,42) = 0.63, *p* = .54, *BF*_incl_ = 0.26, nor for proportions of correct responses, *F*(2,42) = 1.95, *p* = .16, *BF*_incl_ = 0.79. Evidence speaking in favor of these null hypotheses is, however, moderate or anecdotal.

Supplementary analyses of ocular and manual responses to the single-face presentations, performed only on trials that did not contain any misidentified face, came to the same conclusions (see OSM, Section 1.1).

#### Classification analyses

The results of the within-participants classification analyses are summarized in Table 5. In line with our hypothesis, for each group of participants, mean ROC AUCs (all equal to 1), mean balanced accuracies (range: .94–.98) as well as mean F1 scores (range: .87–.96) were all higher than baseline values, all *p*s < .001, all Cohen’s *d*s > 2.5. Then, we performed one-way ANOVAs to test the effect of instructions on mean balanced accuracies and F1 scores (unregistered analyses). The results suggest that the factor Group had no influence on balanced accuracies, *F*(2,42) < 0.42, *p* = .658, or on F1 scores, *F*(2,42) = 1.12, *p* = .333, but it was moderately or not supported by the *BF*_10_ (respectively, 0.22 and 0.36). ANOVA was not performed for the ROC AUCs due to the lack of variance in the corresponding values.

**Table 5:** Results of the within-participants classification analyses for each group of participants in Experiment 1

	Control			Concealment			Simple countermeasure		
	Mean (± *SD*s)	*t*(14)	Cohen’s *d*	Mean (± *SD*s)	*t*(14)	Cohen’s *d*	Mean (± *SD*s)	*t*(14)	Cohen’s *d*
ROC AUC	1 (0)	10.70**	4.04	1 (0)	9.82**	3.71	1 (0)	6.69**	2.53
Acc.	.96 (0.08)	20.55**	7.77	.94 (0.13)	12.47**	4.71	.98 (0.06)	28.02**	10.59
F1 score	.94 (0.12)	29.64**	11.20	.87 (0.26)	12.74**	4.81	.96 (0.09)	38.45**	14.53

Acc. = Balanced accuracy; * = *p* < .05; ** = *p* < .001

As hypothesized, for the between-participants analyses, mean ROC AUCs (range: .93–1), mean balanced accuracies (range: .89–.98) as well as mean F1 scores (range: .83–.97) were all higher than baseline values in all groups of participants, all *p*s < .001, all Cohen’s *d*s > 2.4 (see Table 6). The factor Group influenced ROC AUCs, *F*(2,42) = 17.01, *p* < .001, η^2^_p_ = 0.45, balanced accuracies, *F*(2,42) = 5.06, *p* = 0.011, η^2^_p_ = 0.194, as well as F1 scores, *F*(2,42) = 5.37, *p* = .008, η^2^_p_ = 0.2. The Concealment group had the lowest values, all *p*s < .012, all Cohen’s *d*s > 1.12. However, as all these measures are very high, between-group comparisons are not very informative (unregistered analyses).

**Table 6:** Results of the between-participants classification analyses for each group of participants in Experiment 1

	Control			Concealment			Simple countermeasure		
	Mean (± *SD*s)	*t*(14)	Cohen’s *d*	Mean (± *SD*s)	*t*(14)	Cohen’s *d*	_Mean_ (± *SD*s)	*t*(14)	Cohen’s *d*
ROC AUC	1 (0)	10.63**	4.02	.93 (0.07)	6.45**	2.44	1 (0)	10.60**	4.01
Acc.	.98 (0.03)	51.45**	19.45	.89 (0.09)	16.32**	6.17	.96 (0.09)	15.68**	5.93
F1 score	.97 (0.03)	46.96**	17.45	.83 (0.13)	19.96**	7.54	.93 (0.15)	15.29**	5.78

Acc. = Balanced accuracy; * = *p* < .05; ** = *p* < .001

### Interim discussion

Lancry-Dayan et al.’s (2018) results obtained with faces of acquaintances were reproduced with celebrity faces. Most importantly, between-group differences did not reach significance, neither in their orienting responses nor in their avoidance of famous faces (although evidence for these null hypotheses is moderate). Even participants in the Simple countermeasure group could not thwart this test, which corroborated the observation that the STM-CIT seems resistant to countermeasures. Furthermore, classification analyses performed within and between subjects were able to distinguish familiar from unknown faces with high accuracy, irrespective of the instructions provided to the participants. Consequently, these results show an interesting potential of the STM-CIT to detect concealed familiarity.

In a second experiment, we tested the robustness of these countermeasures by proposing in addition to instructions some explanations for the expected patterns or some feedback on participants’ oculomotor behavior. It is indeed plausible that the instructions given to the countermeasure group were not detailed enough. Consequently, participants may have prioritized the memory task, at the expense of the oculomotor task. In particular, we hypothesized that more precise explanations on their expected gaze allocation would enable participants to reduce their avoidance of the familiar faces. In addition, if instructions had no effect in the previous experiment due to their lack of precision, we should not observe any decrease in manual response times or in the proportion of correct responses. By contrast, a decrease in the memory task performance could indicate a lack of volitional gaze control by the participants.

## Experiment 2

Thirty participants were assigned to two different countermeasure groups that received more precise instructions than in the previous experiment. These groups differed according to the feedback they received during the test. Immediately after signing the consent form, each participant watched a 3-min slideshow (which can be found in our OSF folder) that gave them the same instructions as in the Simple countermeasure group of Experiment 1, and in addition emphasized the importance of both the memory and the eye-movement tasks. In this slideshow, 70 s were dedicated to the presentation of Lancry-Dayan et al.’s (2018) Figure 2, showing the time course of gaze position during the four-face displays in their concealed, non-concealed, and countermeasure groups. After half of the STM-CIT trials, participants of the *Enhanced countermeasure* group were presented with Lancry-Dyan et al.'s (2018) Figure 2 again. Participants of the *Feedback* group were shown a graph similar to those of Lancry-Dayan et al. (2018), but showing the time course of their own gaze position averaged over their first 32 trials. The *Simple countermeasure* group of Experiment 1 was used as a countermeasure baseline group, as participants in this group were given the most succinct explanations of how to conceal their familiarity and thwart the test.

### Results

#### Eye movements during the four-face parallel displays

**Fig. 5:**
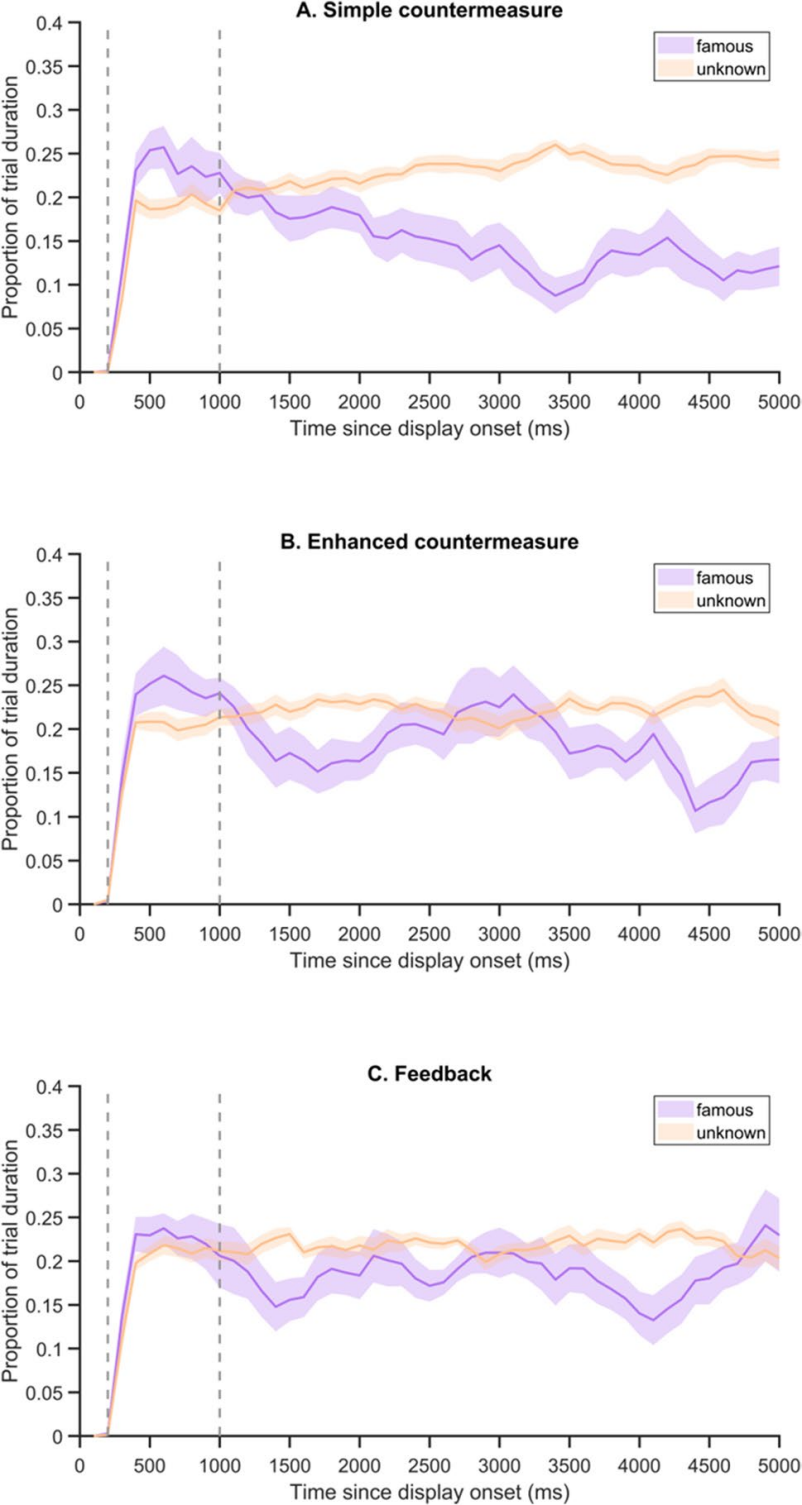
Time course of gaze position for (**A**) the Simple countermeasure group, (**B**) the Enhanced countermeasure, and (**C**) the Feedback groups of Experiment 2, in the last 32 trials of the STM-CIT session. Proportion of time spent on famous vs. unknown faces during the first phase (200–1,000 ms) and second phase (1,001–5,000 ms) of the four-face parallel displays. Time spent on the unknown faces of a trial was averaged across the three pictures. Shadowed areas indicate ± SEM across participants, and dashed lines the beginning of each trial phase (color figure online)

The pre-registered analyses conducted on the 64 STM-CIT trials, similar to the analyses conducted in the first experiment and detailed in the OSM (Section 2), indicated that between-group differences emerged in this second experiment. These differences might be further accentuated during the second part of the STM-CIT, i.e., after the second presentation of Lancry-Dayan et al.’s (2018) results (Enhanced countermeasure group) or after individualized feedback (Feedback group). To better account for the effect of these new instructions, we performed additional unregistered analyses, in which we compared the eye movements made by participants in each group over the last 32 trials. Figure 5 presents the proportions of time spent on famous versus unknown faces, computed over these trials. The overlap between the two curves was particularly evident during the first trial phase for the Feedback group, whereas the orientation-avoidance pattern remained clearly visible for the Simple countermeasure group.

**Fig. 6:**
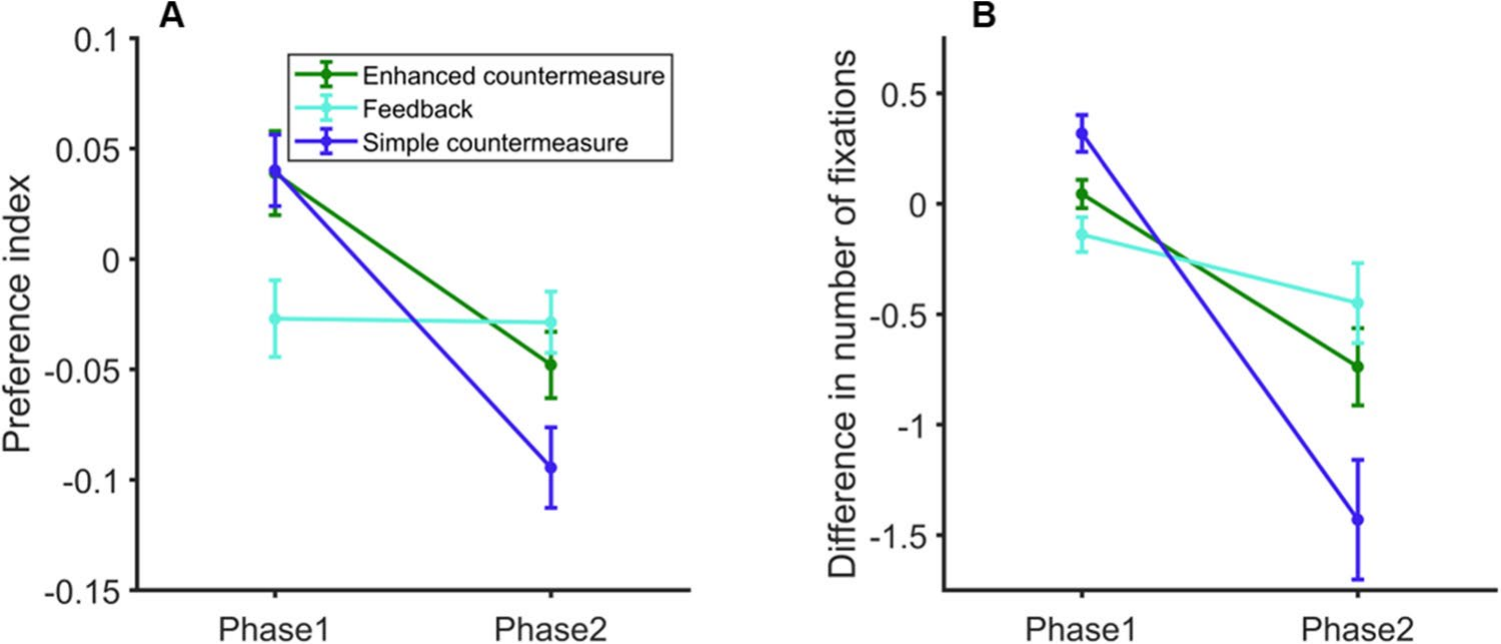
Results of the last 32 STM-CIT trials of Experiment 2. (**A**) Mean preference indices and (**B**) differences between the mean number of fixations on famous vs. unknown faces, obtained for each group, in each phase of the four-face parallel displays. Error bars: SEM (color figure online)

The two mixed ANOVAs confirmed that between-group differences increased in the second part of the STM-CIT (Fig. 6). Most importantly, the interaction between Phase and Group became significant, both for preference indices, *F*(2,42) = 8.12, *p* = .001, η^2^_p_ = 0.28, *BF*_10_ = 165.3, and for the differences between numbers of fixations on famous faces versus unknown faces, *F*(2,42) =10.44, *p* < .001, η^2^_p_ = 0.33, *BF*_10_ = 1078.6. The main effects of Group were not significant, *p*_*s*_ = .29 and .24, although evidence for these null hypotheses was moderate, *BF*_10_ = 0.21 and 0.20, respectively.

The statistical conclusions about the simple effects of Phase were similar for both dependent variables, but differed between groups. In the Feedback group, the trial phase had no effect on preference indices (Mean difference between phases = 0.002, *SD* = 0.08, Tukey post hoc test: *p =* 1), or on numbers of fixations on famous versus unknown faces (Mean difference between phases = 0.31, *SD* = 0.75, Tukey post hoc test: *p =* .75). In contrast, all other differences between phases were significant (all *p*s < .015, all Cohen’s *d*s *>*1.26).

When statistical analyses excluded trials in which a misidentified face had been presented (i.e., when data include only trials in which the faces’ status was totally unambiguous), the conclusions did not change, except that the effect of Group on numbers of fixations on famous versus unknown faces was significant (see OSM, Section 1.3, for more details).

#### Ocular and manual responses in the single-face displays

**Fig. 7:**
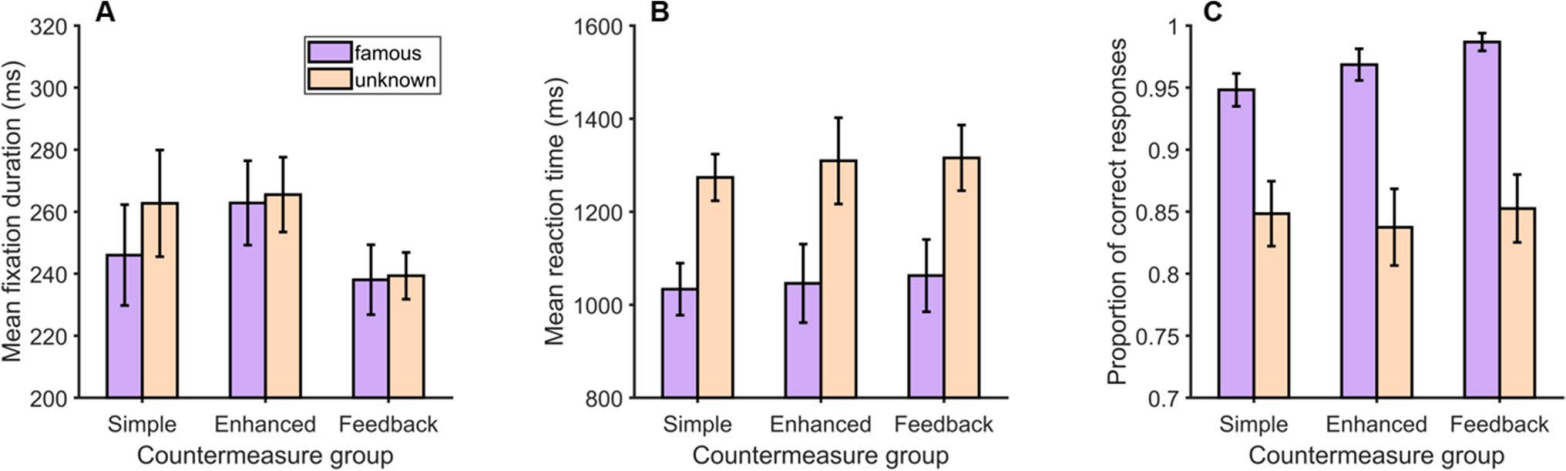
Ocular and manual responses in the single-face displays of the last 32 STM-CIT trials in Experiment 2. (**A**) Mean fixation durations, (**B**) mean reaction times, and (**C**) mean proportions of correct responses in the short-term memory task for famous and unknown faces, for each group of participants. Error bars: SEM (color figure online)

The data obtained on the last 32 trials did not support the presence or absence of an effect of Face on mean fixation durations (*M*_*DurFamous*_ = 249 ms, *SD =* 53.5 ms, *M*_*DurUnknown*_ = 255.9 ms, *SD =* 50.2 ms), *F*(1,42) = 1.62, *p* = .21, *BF*_10_ = 1.03 (see Fig. 7A). The same applied to the main effect of Group, *F*(2,42) = 1.05, *p* = .36, *BF*_10_ = 0.57, and there was moderate evidence for the absence of an interaction between the two factors, *F*(2,42) = 0.82, *p* = .45, *BF*_10_ = 0.28.

As in the first experiment, participants responded faster (Fig. 7B), *F*(1,42) = 60.58, *p* < .001, η^2^_p_ = 0.59, and more accurately (Fig. 7C), *F*(1,42) = 45.2, *p* < .001, η^2^_p_ = 0.52, when a famous face (*M*_*ReactionTime*_ *=* 1,047.3 ms, *SD =* 278.9 ms, *M*_*Proportion_correct*_ *=* 0.97, *SD =* 0.05) than when an unknown face (*M*_*ReactionTime*_ *=* 1,299.6 ms, *SD =* 277.7 ms, *M*_*Proportion_correct*_ *=* 0.85, *SD =* 0.11) was presented in the single-face display. The factor Group did not affect reaction times, *F*(2,42) = 0.07, *p* = .93, *BF*_10_ = 0.2, or the proportions of correct responses, *F*(2,42) = 0.58, *p* = .57, *BF*_10_ = 0.16. Interactions between the two aforementioned factors were not significant, neither for response times, *F*(2,42) = 0.04, *p* = .96, *BF*_incl_ = 0.16, nor for proportions of correct responses, *F*(2,42) = 0.37, *p* = .69, *BF*_incl_ = 0.22.

As in Experiment 1, supplementary analyses of ocular and manual responses to the single-face presentations, conducted after excluding trials that contained misidentified faces, also came to the same conclusions (see OSM, Section 1.3). Finally, restricting the analyses of ocular and manual responses to the last 32 STM-CIT trials yielded the same results as the analyses of the entire session, regarding the non-significant interactions between Phase and Group (see OSM, Section 2, for more details).

#### Classification analyses

Table 7 presents the results of the within-participants classification analyses performed on the last 32 trials of the STM-CIT sessions. As in Experiment 1, for each group of participants, mean ROC AUCs (range: .96–1), mean balanced accuracies (range: .81–.91) as well as mean F1 scores (range: .62–.86) were all higher than baseline values, all *p*s < .001, all Cohen’s *d*s > 1.9. Our data revealed inconclusive evidence regarding the absence of a main effect of Group on these measures, all *F*_*s*_(2,42) < 2.16, all *p*s > .127, *BF*_10_ range: 0.42–0.73 (unregistered analyses). These results were consistent with the classification analyses conducted on the 64 trials (OSM, Section 2).

**Table 7:** Results of the within-participants classification analyses for each group of participants in Experiment 2, obtained over the last 32 trials

	Simple countermeasure			Enhanced countermeasure			Feedback		
	Mean (± *SD*s)	*t*(14)	Cohen’s *d*	Mean (± *SD*s)	*t*(14)	Cohen’s *d*	Mean (± *SD*s)	*t*(14)	Cohen’s *d*
ROC AUC	.96 (.08)	4.95**	1.87	1 (0.01)	10.44**	3.95	.99 (0.03)	7.51**	2.84
Acc.	.81 (.23)	5.18**	1.96	.90 (0.15)	9.82**	3.71	.91 (0.15)	10.14**	3.83
F1 score	.62 (.45)	5.17**	1.95	.84 (0.27)	11.77**	4.45	.86 (0.28)	11.52**	4.35

Acc. = Balanced accuracy; * = *p* < .05; ** = *p* < .001

The between-participants analyses (Table 8) revealed that mean ROC AUCs (range: .82–1), mean balanced accuracies (range: .94–1) and mean F1 scores (range: .73–.99) were all higher than baseline values, all *p*_*s*_ < .001, all *Cohen’s d*_*s*_ > 4.6. The factor Group influenced ROC AUCs, *F*(2,42) = 38.69, *p* < .001, η^2^_p_ = .65, balanced accuracies, *F*(2,42) = 56.54, *p* < 0.001, η^2^ = .73, as well as F1 scores, *F*(2,42) = 52.07, *p* < 0.001, η^2^_p_ = .71 (the data obtained from the 64 trials did not support such a conclusion). The Simple countermeasure group had the lowest values, all *p*s < .001, all Cohen’s *d*s > 2.78. But most importantly, as in Experiment 1, these values were all very high.

**Table 8:** Results of the between-participants classification analyses for each group of participants in Experiment 2, obtained over the last 32 trials

	Simple countermeasure			Enhanced countermeasure			Feedback		
	Mean (± *SD*s)	*t*(14)	Cohen’s *d*	Mean (± *SD*s)	*t*(14)	Cohen’s *d*	Mean (± *SD*s)	*t*(14)	Cohen’s *d*
ROC AUC	.82 (0.08)	15.23**	5.76	.99 (0.03)	68.96**	26.07	1 (0.02)	19.49**	7.37
Acc.	.94 (0.04)	12.37**	4.67	1 (0)	12.79**	4.38	1 (0)	8.33**	3.15
F1 score	.73 (0.13)	19.38**	7.33	.98 (0.03)	115.20**	43.54	.99 (0.02)	15.28**	5.77

Acc. = Balanced accuracy; * = *p* < .05; ** = *p* < .001

### Interim discussion

In line with our hypotheses, the interaction between Phase and Group on the preference indices reached significance in this experiment. Eye movement data obtained over the second half of the STM-CIT trials revealed decisive evidence for this interaction, both on preference indices (η^2^_p_ = 0.28, *BF*_incl_ = 165.3), and on the difference between numbers of fixations on famous vs. unknown faces (η^2^_p_ = 0.33, *BF*_incl_ = 1078.6). This effect was mainly due to the large changes observed over the two trial phases in the Simple countermeasure group, and to a lesser extent, in the Enhanced countermeasure group, compared to the Feedback group. Participants in the latter group were the most capable of controlling their gaze, which suggests that feedback on one’s own performance might help to thwart the STM-CIT.

## General discussion

Lancry-Dayan et al. (2018) proposed a CIT protocol that included a short-term memory task. This STM-CIT appeared to be relevant to identify knowledge about photos of acquaintances even when participants were asked to conceal their familiarity. We reproduced previous studies on STM-CITs with photos of celebrities instead of acquaintances (Lancry-Dayan et al., 2018) or personally familiar objects (Lancry-Dayan et al., 2021). Like these authors, we observed an orienting response towards familiar faces during the first second of their presentation, followed by avoidance of these faces.

In Experiment 1, orienting-avoidance patterns were observed in the Control, Concealment, and Simple countermeasure groups. In Experiment 2, our data provided decisive evidence for an interaction between phases and instructions in the second half of the STM-CIT trials (following the new exchange with the experimenter in the Enhanced countermeasure and Feedback groups). In these trials, the orientation-avoidance pattern disappeared only when participants were given precise individual feedback on their own oculomotor performance (Feedback group). By contrast, this pattern was still visible for the two other countermeasure groups (see Fig. 6), which was consistent with Lancry-Dayan et al.’s (2018, 2021) results. These authors interpreted such a difficulty in controlling eye movements, in particular during the second trial phase, as a conflict between the demands of the memory task and high-level attempts to follow the concealment instructions. The present study shows that feedback on individual oculomotor performance helped participants to deal with this conflict and to reduce the difference between eye movements made towards famous and unknown faces.

Despite the disappearance of the orienting-avoidance pattern in the Feedback group, classification analyses could still be used to distinguish between familiar and unfamiliar faces with high accuracy. The classification performance was even higher than that obtained in the Simple countermeasure group (this slight difference, which contrasts with the ANOVA findings, may be due to the combination of several measures in the classification analyses). As in Lancry-Dayan et al. (2018), we simulated a sample of unknowledgeable observers (non-existent in our study but crucial in applied contexts) by utilizing the trials in which a famous face did not appear. These trials were used not only to differentiate between critical (famous faces) and irrelevant items (unknown faces), but also to compute baseline classification performance. The levels of classification efficiency we obtained are consistent with those obtained by Lancry-Dayan et al. (2018, 2021) on STM-CITs, and are in the upper range of the ROC AUC values reported in the classic CIT literature (Ben-Shakhar, 2011; Meijer et al., 2014).

In line with Lancry-Dayan et al. (2018), we expected longer fixations on famous faces presented in the single-face displays than on unknown faces, due to a familiarity effect (Althoff & Cohen, 1999; Heisz & Shore, 2008; Ryan et al., 2007; Schwedes & Wentura, 2012). The fixation durations observed in our study were, however, inconsistent with this assumption. In Experiment 1, they were not affected by the type of face, while in Experiment 2, we observed longer fixations on unknown faces than on famous faces, and data obtained over the last 32 trials revealed inconclusive evidence. It is possible that participants continued to follow the countermeasure instructions (whatever their degree of precision) also during single face presentation. In trying to modify their eye movements, they deliberately scanned the famous faces more quickly.

Regarding memory-task performances, like Lancry-Dayan et al. (2018, 2021), we observed shorter response times and higher proportions of correct responses when celebrity faces were presented. Together with the orienting-avoidance pattern, these findings likely reflected an efficient encoding process for celebrity faces, whose representations already existed in long-term memory. Most importantly, the high performance observed in the five groups of participants suggests that all of them obtained enough information during the parallel displays to perform the short-term memory task correctly, irrespective of whether they received detailed explanations about their expected gaze allocation. Hence, memory task performance cannot be used as an indicator of a possible intention to thwart the STM-CIT.

The present study suggests that providing individual feedback is an effective method for training participants to thwart this test and that the STM-CIT associated with classification analyses could constitute a reliable and efficient tool to detect concealed familiarity, even in the strictest countermeasure group. Nevertheless, it is highly unlikely that guilty observers would receive the same in-depth training as the participants in our second experiment. In contrast, the countermeasure method employed by Lancry-Dayan et al. (2021, Experiment 3), consisting in instructing participants to fixate equally and sequentially on all stimuli, was simpler and can be more easily applied in practice. This method also significantly attenuated the differences between eye movements made towards familiar and unfamiliar faces. Notably, the authors also obtained high detection efficiency, even with such instructions. Together with the present study, these findings suggest that the STM-CIT is less vulnerable to countermeasures than the classic CIT (Ben-Shakhar, 2011). Such results are especially interesting for applied purposes, and are of high importance in the CIT literature where the tradition is to evaluate the efficiency of the CIT and its variants in identifying recognized (crime-related) information. In particular, recent research demonstrated the potential of CITs based on event-related potential for subverting countermeasures (Rosenfeld, 2019; Zheng et al., 2022). However, in applied contexts, ERP methods are difficult to implement. In comparison, the STM-CIT, which does not require the attachment of sensors or electrodes, seems easier to use. For these reasons, the STM-CIT might be a promising tool for detecting concealed information in forensic investigations.

## Conclusion

We reproduced Lancry-Dayan et al.’s (2018, 2021) studies on STM-CIT by using celebrity faces as familiar items. As in the seminal study, we found orientation-avoidance ocular patterns in each of the three groups of participants (Control, Concealment, and Simple countermeasure) who were given one of the instructions provided by Lancry-Dayan et al. (2018). In contrast, one way to balance the time spent on each face was to provide feedback on individuals’ oculomotor behavior in addition to detailed explanation about the expected results - which is unlikely to occur in applied contexts. These findings suggest that the inclusion of a memory task in the CIT enhances differences between eye movements made towards familiar versus unfamiliar faces, at least to a certain extent. Together with previous work (Lancry-Dayan et al., 2018, 2021), the present study shows that the contribution of classification analyses further increases the STM-CIT power to detect concealed familiarity.

### Supplementary information

ESM 1 (DOCX 413 kb)

## Footnotes

1 This number was determined following an error in our a priori power analysis. In a second analysis, we found that 17 participants would have been sufficient to reliably detect an interaction of the same effect size as the one observed by Lancry-Dayan et al. (2018, Supplementary Material) in their omnibus ANOVA on dwell-time measures, with two within-subjects factors (Type of faces: familiar vs. unfamiliar, and Dwell time phase: first vs. second) and one between-subjects factor (their three experiments): *F*(2,84) = 9.489, *p* < .001, η^2^_p_ = 0.184. In the present study, the calculation of a preference index reduced the number of measurements to two (one per trial phase). The desired power was set to 0.8, alpha to .05 and the correlation was factored into the effect size.
