# Supplementary material for: Resistance of a short-term memory concealed information test with famous faces to countermeasures
Source: Mem Cognit. 2023 Dec 5;52(3):632–47. doi: 10.3758/s13421-023-01489-1 (PMC11021291; doi:10.3758/s13421-023-01489-1)
Supplement: Supplementary file 1 — (DOCX 413 kb) [file 13421_2023_1489_MOESM1_ESM.docx]

**SUPPLEMENTARY MATERIALS**

Resistance of a Short-term Memory Concealed Information Test

with Famous Faces to Countermeasures

Hugues Delmas, Camélia Ciocan, Mariya Novopashyna, & Céline Paeye

**Table of contents**

[**1. Analyses without misidentification trials 2**](#_heading=h.2afjlb9d9ugm)

[1.1. Experiment 1 2](#_heading=h.iu3kr0ij5n66)

[1.1.1. Eye movements during the four-face parallel displays 2](#_heading=h.kgp6s51bq53j)

[1.1.2. Ocular and manual responses in the single face displays 4](#_heading=h.vf8sj5k9j4dr)

[1.2. Experiment 2 - 64 trials 6](#_heading=h.wqy7ef57vjoj)

[1.2.1. Eye movements during the four-face parallel displays 6](#_heading=h.8kr76zpzfq9y)

[1.2.2. Ocular and manual responses in the single face displays 7](#_heading=h.ibwobj1b3ywj)

[1.3. Experiment 2 - 32 trials 10](#_heading=h.a69bm69tfamg)

[1.3.1. Eye movements during the four-face parallel displays 10](#_heading=h.xtogbxegl9aw)

[1.3.2. Ocular and manual responses in the single face displays 11](#_heading=h.oir65ebdp71c)

[**2. Analyses of the 64 STM-CIT trials of Experiment 2 13**](#_heading=)

[2.1. Eye movements during the four-face parallel displays 13](#_heading=h.kz5zeaq6z50h)

[2.2. Ocular and manual responses in the single face displays 15](#_heading=h.aapzdtddlmi1)

[2.3. Classification analyses 16](#_heading=h.dc0ha0tn8br2)

# Analyses without misidentification trials

The analyses reported in this first supplementary materials’ section are the same as those presented in the main text, except that we have excluded all the misidentification trials (*false recognition* trials, 0.81% of the trials, see Table 3 in the main text, and *miss* trials, on average, 6.31% of the trials) in order to avoid any ambiguity in the interpretation of faces’ status.

This led us to remove one participant of the Concealment group and one participant of the Simple countermeasure group from the parallel display analyses due to a high miss rate (*z*-score > 3; more than 25% of miss trials, see Lancry-Dayan et al., 2021). As only parallel displays containing a famous face were considered in these analyses, the three participants who had a high false recognition rate (*z*-score > 3; more than 50% of false recognition trials) but a low miss rate, were still included in these analyses. These participants (one from the Control group and two from the Simple countermeasure group) were removed from the single display and classification analyses. After the removal of these participants, miss and false recognition trials accounted respectively for 0.49% (*SD* = 1.63) and 3.51% (*SD* = 7.07) of the 64 STM-CIT trials, on average.

## 1.1. Experiment 1

### *1.1.1. Eye movements during the four-face parallel displays*

Preference indices

Mean preference indices (± *SD*) for each group of participants

| Group | Phase 1 | Phase 2 |
| --- | --- | --- |
| Control | 0.04 (± 0.05) | -0.13 (± 0.05) |
| Concealment | 0.05 (± 0.07) | -0.10 (± 0.03) |
| Simple countermeasure | 0.03 (± 0.06) | -0.09 (±0.08) |

Mixed ANOVA with one between-subjects factor (Group) and one within-subjects factor (Phase)

| Effect | *F* | df | *p* | η²_p_ or *BF* |
| --- | --- | --- | --- | --- |
| Phase | 146.7 | 1,40 | < .001 | η²_p_ = 0.79 |
| Group | 0.87 | 2,40 | .428 | *BF*_10_ = 0.15 |
| Phase * Group | 1.74 | 2,40 | .189 | *BF*_incl_ = 0.70 |

The statistical conclusions are similar to those obtained from the trials that included face misidentifications, except that the level of evidence for the interaction here was anecdotal.

Differences in number of fixations

Mean differences (± *SD*) between the number of fixations on famous vs. unknown faces for each group of participants

| Group | Phase 1 | Phase 2 |
| --- | --- | --- |
| Control | 0.18 (± 0.27) | -1.97 (± 0.92) |
| Concealment | 0.20 (± 0.24) | -1.41 (± 0.54) |
| Simple countermeasure | 0.14 (± 0.29) | -1.48 (± 1.26) |

Mixed ANOVA with one between-subjects factor (Group) and one within-subjects factor (Phase)

| Effect | *F* | df | *p* | η²_p_ or *BF* |
| --- | --- | --- | --- | --- |
| Phase | 136.9 | 1,40 | < .001 | η²_p_ = 0.77 |
| Group | 1.42 | 2,40 | .254 | *BF*_10_ = 0.17 |
| Phase * Group | 1.40 | 2,40 | .259 | *BF*_incl_ = 0.57 |

The statistical conclusions are similar to those obtained from the trials that included face misidentifications, except that the level of evidence for the interaction here was anecdotal.

### *1.1.2. Ocular and manual responses in the single face displays*

Mean fixation duration

Mean fixation durations (in ms, ± *SD*) for famous and unknown faces, for each group of participants

| Group | Famous | Unknown |
| --- | --- | --- |
| Control | 252.5 (± 56.7) | 256.9 (± 52) |
| Concealment | 290.9 (± 77.6) | 288.1 (± 84.3) |
| Simple countermeasure | 234.6 (± 38.7) | 243.2 (± 25.8) |

Mixed ANOVA with one between-subjects factor (Group) and one within-subjects factor (Face)

| Effect | *F* | df | *p* | η²_p_ or *BF* |
| --- | --- | --- | --- | --- |
| Face | 0.64 | 1,37 | .428 | *BF*_10_ = 0.29 |
| Group | 2.55 | 2,37 | .092 | *BF*_10_ = 0.82 |
| Face * Group | 0.59 | 2,37 | .559 | *BF*_incl_ = 0.23 |

The statistical conclusions are similar to those obtained from the trials that included face misidentifications.

Mean reaction times in the short-term memory task

Mean reaction times (in ms, ± *SD*) for famous and unknown faces, for each group of participants

| Group | Famous | Unknown |
| --- | --- | --- |
| Control | 958.5 (± 214.6) | 1222.7 (± 211.8) |
| Concealment | 1023.9 (± 175.6) | 1345 (± 172.3) |
| Simple countermeasure | 1055 (± 192.3) | 1360.5 (± 282.5) |

Mixed ANOVA with one between-subjects factor (Group) and one within-subjects factor (Face)

| Effect | *F* | df | *p* | η²_p_ or *BF* |
| --- | --- | --- | --- | --- |
| Face | 146 | 1,37 | < .001 | η²_p_ = 0.8 |
| Group | 1.36 | 2,37 | .27 | *BF*_10_ = 0.37 |
| Face * Group | 0.50 | 2,37 | .61 | *BF*_incl_ = 0.25 |

The statistical conclusions are similar to those obtained from the trials that included face misidentifications.

Mean proportions of correct responses in the short-term memory task

Mean percentages of correct responses for famous and unknown faces, for each group of participants.

| Group | Famous | Unknown |
| --- | --- | --- |
| Control | 0.97 (± 0.04) | 0.89 (± 0.05) |
| Concealment | 0.98 (± 0.03) | 0.85 (± 0.06) |
| Simple countermeasure | 0.96 (± 0.03) | 0.86 (± 0.08) |

Mixed ANOVA with one between-subjects factor (Group) and one within-subjects factor (Face)

| Effect | *F* | df | *p* | η²_p_ or *BF* |
| --- | --- | --- | --- | --- |
| Face | 98.88 | 1,37 | < .001 | η²_p_ = 0.73 |
| Group | 0.97 | 2,37 | .387 | *BF*_10_ = 0.37 |
| Face * Group | 1.31 | 2,37 | .281 | *BF*_incl_ = 0.24 |

The statistical conclusions are similar to those obtained from the trials that included face misidentifications.

## 1.2. Experiment 2 - 64 trials

### *1.2.1. Eye movements during the four-face parallel displays*

Preference indices

Mean preference indices (± *SD*) for each group of participants

| Group | Phase 1 | Phase 2 |
| --- | --- | --- |
| Simple countermeasure | 0.03 (± 0.06) | -0.09 (± 0.09)** |
| Enhanced countermeasure | 0.01 (± 0.05) | -0.05 (± 0.06)** |
| Feedback | 0.02 (± 0.06) | -0.04 (±0.05)** |

** p < .05 and ** p < .01 for one-sample t-test (or Wilcoxon signed-rank test) comparing the preference indices of each phase against the zero value*

Mixed ANOVA with one between-subjects factor (Group) and one within-subjects factor (Phase)

| Effect | *F* | df | *p* | η²_p_ or *BF* |
| --- | --- | --- | --- | --- |
| Phase | 48.83 | 1,41 | < .001 | η²_p_ = 0.54 |
| Group | 0.96 | 2,41 | .393 | *BF*_10_ = 0.2 |
| Phase * Group | 2.48 | 2,41 | .096 | *BF*_incl_ = 1.17 |

The statistical conclusions are similar to those obtained from the trials that included face misidentifications, except that there was no evidence for the interaction between Phase and Group. All preference indices in Phase 2 are significantly lower than zero (all *p_s_* < .006).

Differences in number of fixations

Mean differences (± *SD*) between the number of fixations on famous vs. unknown faces for each group of participants

| Group | Phase 1 | Phase 2 |
| --- | --- | --- |
| Simple countermeasure | 0.14 (± 0.29) | -1.49 (± 1.26)** |
| Enhanced countermeasure | -0.01 (± 0.17) | -0.73 (± 0.76)** |
| Feedback | 0.08 (± 0.26) | -0.61 (± 0.73)** |

** p < .05 and ** p < .01 for one-sample t-test (or Wilcoxon signed-rank test) comparing the preference indices of each phase against the zero value*

Mixed ANOVA with one between-subjects factor (Group) and one within-subjects factor (Phase)

| Effect | *F* | df | *p* | η²_p_ or *BF* |
| --- | --- | --- | --- | --- |
| Phase | 51.19 | 1,41 | < .001 | η²_p_ = 0.55 |
| Group | 2.59 | 2,41 | .087 | *BF*_10_ = 0.36 |
| Phase * Group | 4.51 | 2,41 | .017 | η²_p_ = 0.18 |

Tukey post-hoc tests for within-group comparisons (mean differences between Phase 1 and Phase 2)

| Group | Mean difference | *t* | *p* | Cohen’s *d* |
| --- | --- | --- | --- | --- |
| Simple countermeasure | 1.62 | 6.46 | < .001 | 2.36 |
| Enhanced countermeasure | 0.72 | 2.97 | .052 | 0.04 |
| Feedback | 0.70 | 2.89 | .065 | 1.02 |

As for the entire set of trials that included face misidentifications, we found a main effect of Phase. There was anecdotal evidence for an absence of difference between groups. More strikingly, the interaction between Phase and Group reached significance (*BF*_incl_ = 6.47). This interaction was mainly explained by the significant difference between phases observed in the Simple countermeasure group: this difference is more than twice that observed in the other two groups.

### *1.2.2. Ocular and manual responses in the single face displays*

Mean fixation duration

Mean fixation durations (in ms, ± *SD*) for famous and unknown faces, for each group of participants

| Group | Famous | Unknown |
| --- | --- | --- |
| Simple countermeasure | 234.6 (± 38.7) | 243.2 (± 25.8) |
| Enhanced countermeasure | 252.3 (± 61.8) | 269.1 (± 59.4) |
| Feedback | 266.7 (± 46.6) | 269.5 (± 39) |

Mixed ANOVA with one between-subjects factor (Group) and one within-subjects factor (Face)

| Effect | *F* | df | *p* | η²_p_ or *BF* |
| --- | --- | --- | --- | --- |
| Face | 5.37 | 1,39 | .026 | η²_p_ = 0.12 |
| Group | 1.41 | 2,39 | .257 | *BF*_10_ = 0.89 |
| Face * Group | 1.08 | 2,39 | .349 | *BF*_incl_ = 0.35 |

The statistical conclusions are similar to those obtained from the trials that included face misidentifications.

Mean reaction times in the short-term memory task

Mean reaction times (in ms, ± SD) for famous and unknown faces, for each group of participants

| Group | Famous | Unknown |
| --- | --- | --- |
| Simple countermeasure | 1055 (± 192.3) | 1360.5 (± 282.5) |
| Enhanced countermeasure | 1061.4 (± 266.8) | 1334.5 (± 345.3) |
| Feedback | 1096.3 (± 290.3) | 1359.6 (± 247.3) |

Mixed ANOVA with one between-subjects factor (Group) and one within-subjects factor (Face)

| Effect | *F* | df | *p* | η²_p_ or *BF* |
| --- | --- | --- | --- | --- |
| Face | 115.4 | 1,39 | < .001 | η²_p_ = 0.75 |
| Group | 0.05 | 2,39 | .951 | *BF*_10_ = 0.21 |
| Face * Group | 0.22 | 2,39 | .801 | *BF*_incl_ = 0.22 |

The statistical conclusions are similar to those obtained from the trials that included face misidentifications.

Mean proportions of correct responses in the short-term memory task

Mean percentages of correct responses for famous and unknown faces, for each group of participants.

| Group | Famous | Unknown |
| --- | --- | --- |
| Simple countermeasure | 0.96 (± 0.03) | 0.86 (± 0.08) |
| Enhanced countermeasure | 0.97 (± 0.03) | 0.84 (± 0.09) |
| Feedback | 0.99 (± 0.02) | 0.84 (± 0.08) |

Mixed ANOVA with one between-subjects factor (Group) and one within-subjects factor (Face)

| Effect | *F* | df | *p* | η²_p_ or *BF* |
| --- | --- | --- | --- | --- |
| Face | 96.4 | 1,39 | < .001 | η²_p_ = 0.71 |
| Group | 0.27 | 2,39 | .768 | *BF*_10_ = 0.14 |
| Face * Group | 0.77 | 2,39 | .469 | *BF*_incl_ = 0.32 |

The statistical conclusions are similar to those obtained from the trials that included face misidentifications.

## 1.3. Experiment 2 - 32 trials

### *1.3.1. Eye movements during the four-face parallel displays*

Preference indices

Mean preference indices (± *SD*) for each group of participants

| Group | Phase 1 | Phase 2 |
| --- | --- | --- |
| Simple countermeasure | 0.02 (± 0.08) | -0.12 (± 0.08) ** |
| Enhanced countermeasure | 0.04 (± 0.07) | -0.05 (± 0.06) ** |
| Feedback | -0.03 (± 0.07) | -0.03 (±0.05) |

** p < .05 and ** p < .01 for one-sample t-test (or Wilcoxon signed-rank test) comparing the preference indices of each phase against the zero value*

Mixed ANOVA with one between-subjects factor (Group) and one within-subjects factor (Phase)

| Effect | *F* | df | *p* | η²_p_ or *BF* |
| --- | --- | --- | --- | --- |
| Phase | 29 | 1,41 | < .001 | η²_p_ = 0.41 |
| Group | 2.79 | 2,41 | 0.07 | *BF*_10_ = 0.41 |
| Phase * Group | 8.3 | 2,41 | < .001 | η²_p_ = 0.29 |

Tukey post-hoc tests for within-group comparisons (mean differences between Phase 1 and Phase 2)

| Group | Mean difference | *t* | *p* | Cohen’s *d* |
| --- | --- | --- | --- | --- |
| Simple countermeasure | 0.15 | 5.69 | < .001 | 2.11 |
| Enhanced countermeasure | 0.09 | 3.48 | 0.01 | 1.25 |
| Feedback | 0.002 | 0.07 | 1 |  |

The statistical conclusions are similar to those obtained from the trials that included face misidentifications.

Differences in number of fixations

Mean differences (± *SD*) between the number of fixations on famous vs. unknown faces for each group of participants

| Group | Phase 1 | Phase 2 |
| --- | --- | --- |
| Simple countermeasure | 0.13 (± 0.37) | -1.95 (± 1.25) ** |
| Enhanced countermeasure | 0.04 (± 0.25) | -0.75 (± 0.7) ** |
| Feedback | -0.14 (± 0.31) | -0.45 (± 0.7) * |

** p < .05 and ** p < .01 for one-sample t-test (or Wilcoxon signed-rank test) comparing the preference indices of each phase against the zero value*

Mixed ANOVA with one between-subjects factor (Group) and one within-subjects factor (Phase)

| Effect | *F* | df | *p* | η²_p_ or *BF* |
| --- | --- | --- | --- | --- |
| Phase | 24.72 | 1,41 | < .001 | η²_p_ = 0.56 |
| Group | 7.22 | 2,41 | .002 | η²_p_ = 0.26 |
| Phase * Group | 12.04 | 2,41 | < .001 | η²_p_ = 0.39 |

Tukey post-hoc tests for within-group comparisons (mean differences between Phase 1 and Phase 2)

| Group | Mean difference | *t* | *p* | Cohen’s *d* |
| --- | --- | --- | --- | --- |
| Simple countermeasure | 2.08 | 8.06 | < .001 | 3.06 |
| Enhanced countermeasure | 0.79 | 3.17 | 0.03 | 1.16 |
| Feedback | 0.31 | 1.24 | .81 |  |

The statistical conclusions are similar to those obtained from the trials that included face misidentifications, except that the effect of Group was significant.

### *1.3.2. Ocular and manual responses in the single face displays*

Mean fixation duration

Mean fixation durations (in ms, ± *SD*) for famous and unknown faces, for each group of participants

| Group | Famous | Unknown |
| --- | --- | --- |
| Simple countermeasure | 232.7 (± 40.9) | 237.5 (± 26.3) |
| Enhanced countermeasure | 246 (± 62.9) | 262.7 (± 66.6) |
| Feedback | 262.8 (± 52.7) | 265.5 (± 46.7) |

Mixed ANOVA with one between-subjects factor (Group) and one within-subjects factor (Face)

| Effect | *F* | df | *p* | η²_p_ or *BF* |
| --- | --- | --- | --- | --- |
| Face | 1.98 | 1,39 | .167 | *BF*_10_ = 0.56 |
| Group | 1.2 | 2,39 | .313 | *BF*_10_ = 0.59 |
| Face * Group | 0.62 | 2,39 | .546 | *BF*_incl_ = 0.31 |

The statistical conclusions are similar to those obtained from the trials that included face misidentifications.

Mean reaction times in the short-term memory task

Mean reaction times (in ms, ± SD) for famous and unknown faces, for each group of participants

| Group | Famous | Unknown |
| --- | --- | --- |
| Simple countermeasure | 1059.2 (± 197.9) | 1267.4 (± 204.1) |
| Enhanced countermeasure | 1045.9 (± 326.5) | 1309.5 (± 359) |
| Feedback | 1062.6 (± 301.1) | 1315.6 (± 273.4) |

Mixed ANOVA with one between-subjects factor (Group) and one within-subjects factor (Face)

| Effect | *F* | df | *p* | η²_p_ or *BF* |
| --- | --- | --- | --- | --- |
| Face | 57.1 | 1,39 | < .001 | η²_p_ = 0.59 |
| Group | 0.03 | 2,39 | .97 | *BF*_10_ = 0.21 |
| Face * Group | 0.26 | 2,39 | .769 | *BF*_incl_ = 0.2 |

The statistical conclusions are similar to those obtained from the trials that included face misidentifications.

Mean proportions of correct responses in the short-term memory task

Mean percentages of correct responses for famous and unknown faces, for each group of participants.

| Group | Famous | Unknown |
| --- | --- | --- |
| Simple countermeasure | 0.94 (± 0.05) | 0.87 (± 0.1) |
| Enhanced countermeasure | 0.97 (± 0.05) | 0.84 (± 0.12) |
| Feedback | 0.99 (± 0.03) | 0.85 (± 0.11) |

Mixed ANOVA with one between-subjects factor (Group) and one within-subjects factor (Face)

| Effect | *F* | df | *p* | η²_p_ or *BF* |
| --- | --- | --- | --- | --- |
| Face | 36.9 | 1,39 | < .001 | η²_p_ = 0.49 |
| Group | 0.35 | 2,39 | .706 | *BF*_10_ = 0.15 |
| Face * Group | 1.13 | 2,39 | .333 | *BF*_incl_ = 0.47 |

The statistical conclusions are similar to those obtained from the trials that included face misidentifications, except that the level of evidence for an absence of interaction was anecdotal.

# Analyses of the 64 STM-CIT trials of Experiment 2

## 2.1. Eye movements during the four-face parallel displays

Figure S1 shows the time course analysis of gaze position for the two new countermeasure groups of Experiment 2. In comparison to the Simple countermeasure group of Experiment 1, differences in gaze allocation between famous and unknown faces were attenuated, in both the first (orienting) phase and second (avoidance) phase.


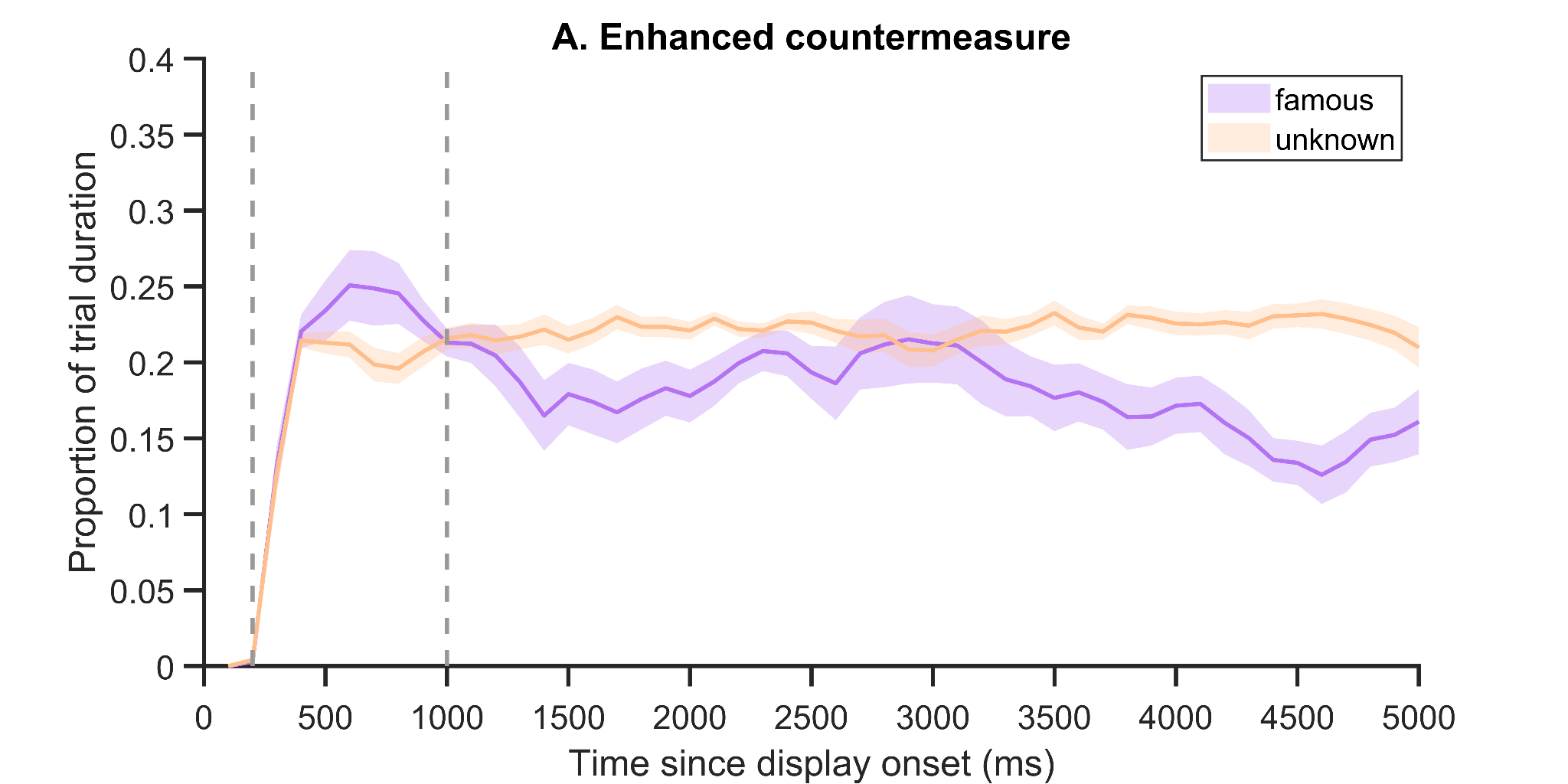

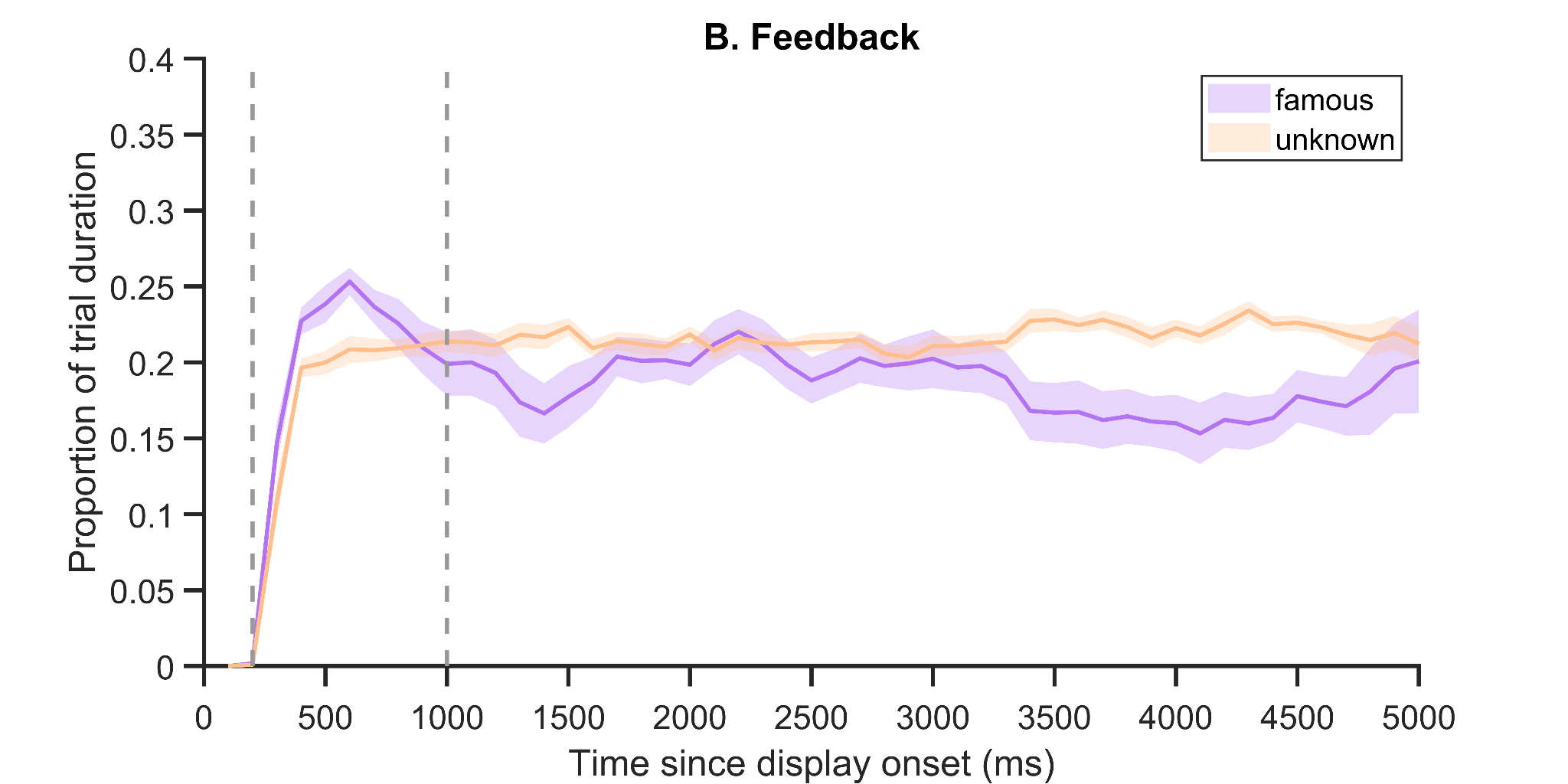


**Figure S1.** Time course of gaze position for **A.** the Enhanced countermeasure and **B.** the Feedback groups of Experiment 2. Proportion of time spent on famous vs. unknown faces during the first phase (200-1000 ms) and second phase (1001-5000 ms) of the four-face parallel displays. Time spent on the unknown faces of a trial was averaged across the three pictures. Shadowed areas indicate ± SEM across participants, and dashed lines the beginning of each trial phase.

The ANOVA confirmed the main effect of Phase on preference indices, *F*(1,42) = 35.91, *p* < .001, η²_p_ = 0.46, with larger mean indices in the first trial phase (*M_Index_ =* 0.02, *SD =* 0.05) than in the second phase (*M_Index_ =* -0.04, *SD =* 0.06). Most importantly, we found a significant interaction between Phase and Group, *F*(2,42) = 5.98, *p* = .005, η²_p_ = 0.22 (see Figure S2A), but no main effect of Group, *F*(2,42) = 0.14, *p* = .87, *BF*_10_ = 0.15 (nevertheless, when trials containing a misidentified face were removed, there was no evidence for the interaction between Phase and Group, see Section 1.2). The interaction effect between Phase and Group is mainly explained by the large difference between phases observed in the Simple countermeasure group (*Mean difference* = 0.11, *SD* = 0.06, Tukey post-hoc test: *p <* .001, *Cohen’s d* = 2.1). This difference was 0.06 (*SD =* 0.11, Tukey post-hoc test: *p* = .03, *Cohen’s d* = 1.09) in the Enhanced countermeasure group and 0.02 (*SD* = 0.04, Tukey post-hoc test: *p* = .84) in the Feedback group (unregistered additional analyses). In addition, for the two new countermeasure groups in this experiment, frequentist *t*-tests indicated that only the preference index observed for the Enhanced countermeasure group in the second phase was significantly smaller than zero, *t*(14) = -2.5, *p* = 0.03, *Cohen’s d* = -0.65. In all other cases, Bayes factors were too close to one (*BF_10_* range: 0.27–2.62) to allow us to draw a strong conclusion for or against a difference from zero (unregistered additional analyses).


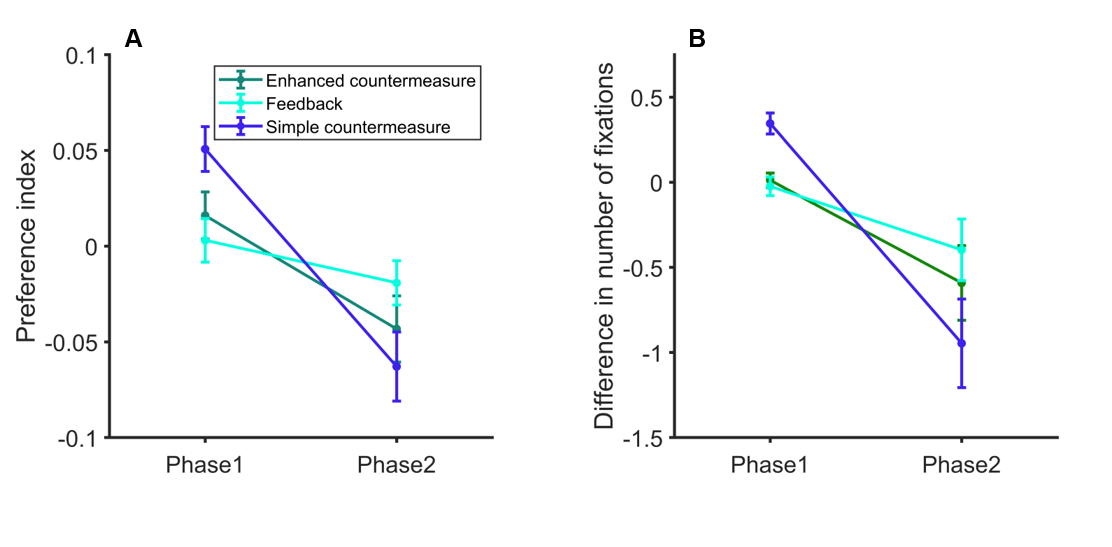


**Figure S2. A.** Mean preference indices and **B.** Differences between the mean number of fixations on famous vs. unknown faces, obtained for each group in Experiment 2, in each phase of the four-face parallel displays. Error bars: SEM.

As shown in Figure S2B, the mean differences between numbers of fixations on famous faces vs. unknown faces changed during the trials, with a positive difference in the first trial phase (*M_deltaFix_* = 0.11, *SD =* 0.26) and a negative, significantly smaller, difference in the second phase (*M_deltaFix_* = -1.82, *SD =* 0.67), *F*(1,42) = 295.9, *p* < .001, η²_p_ = 0.88. The interaction between Phase and Group, however, did not reach significance, *F*(2,42) = 1.29, *p* = .29, but evidence for this null hypothesis is anecdotal, *BF*_incl_ = 0.49, and there was no significant difference between groups *F*(2,42) = 1.92, *p* = 0.16, *BF*_10_ = 0.15. While differences in fixation counts observed in the two new countermeasure groups were not significant in the first phase (*t*-tests against zero, both *p*_s_ > .67, *BF*_10_ = 0.27 and 0.28), they were significantly lower than zero in the second phase (both *p*_s_ < .001, unregistered additional analyses). Note that the interaction between Phase and Group reached significance when trials containing a misidentified face were removed (see Section 1.2). Again, this interaction was explained by the large difference between phases observed in the Simple countermeasure group.

## 2.2. Ocular and manual responses in the single face displays

Contrary to our expectations, participants made longer fixations on unknown faces (*M_Dur_* = 261.9 ms, *SD =* 44 ms) than on famous faces (*M_Dur_* = 253.4 ms, *SD =* 49.2 ms), *F*(1,42) = 5.26, *p* = .027, η²_p_ = 0.11 (Figure S3A). Frequentist statistics (but not Bayesian) indicated that there was no main effect of Group, *F*(2,42) = 1.28, *p* = .29, *BF*_10_ = 0.64, and no interaction, *F*(2,42) = 1.06, *p* = .36, *BF*_incl_ = 0.39.


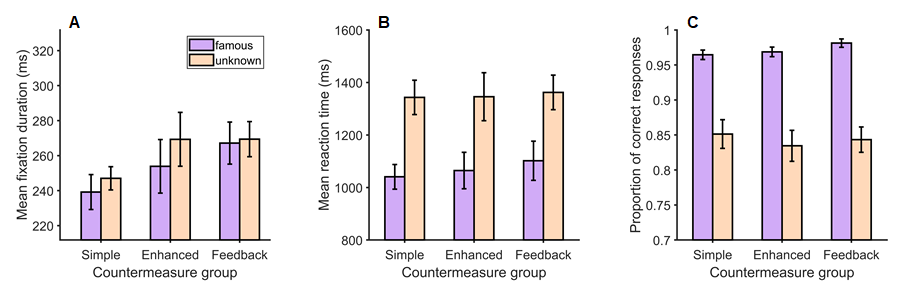


**Figure S3.** Results of the last 32 STM-CIT trials of Experiment 2. **A.** Mean fixation durations during the single displays, **B.** Mean reaction times and **C.** Mean proportions of correct responses in the short-term memory task for famous and unknown faces, for each group of participants. Error bars: SEM.

As in the last 32 trials, participants responded faster (Figure S3B), *F*(1,42) = 126.27, *p* < .001, η²_p_ = 0.75, and more accurately (Figure S3C), *F*(1,42) = 109.5, *p* < .001, η²_p_ = 0.72, when a famous face (*M_ReactionTime_* = 1069 ms, *SD* = 246.6 ms, *M_Proportion_correct_* = 0.97, SD = 0.03) than when an unknown face (*M_ReactionTime_* = 1350.3 ms, *SD* = 254.8 ms, *M_Proportion_correct_* = 0.84, SD = 0.08) was presented in the single face display. The factor Group did not affect reaction times, *F*(2,42) = 0.09, *p* = .91, *BF*_10_ = 0.2, or the proportions of correct responses, *F*(2,42) = 0.25, *p* = .78, *BF*_10_ = 0.13. Interactions between the two aforementioned factors were not significant, neither for response times, *F*(2,42) = 0.24, p = .79, *BF*_incl_ = 0.19, nor for proportions of correct responses, *F*(2,42) = 0.39, *p* = .68, *BF*_incl_ = 0.25. Analyses of ocular and manual responses to the single-face presentations, conducted after excluding misidentification trials, also came to the same conclusions (see Section 1.2).

## 2.3. Classification analyses

For both groups of participants, mean ROC AUCs, mean balanced accuracies and mean F1 scores were higher than baseline values, all *p_s_* < .001, all *Cohen’s d_s_* > 3.67 (Table ST1). To test whether there were differences in classification efficiency between the three countermeasure groups, we performed one-way ANOVAs on ROC AUCs, mean accuracies and F1 scores. Our data revealed inconclusive evidence for the absence of a Group effect on these measures, all *F_s_*(2,42) < 1.14, all *p_s_* > .331, *BF*_10_ range: 0.33–0.36 (unregistered analyses).

Table ST1: Results of the within-participants classification analyses for the two new countermeasure groups in Experiment 2

|  | Enhanced countermeasure | | | Feedback | | |
| --- | --- | --- | --- | --- | --- | --- |
|  | Mean (±*SD*s) | *t*(14) | *Cohen’s d* | Mean (±*SD*s) | *t*(14) | *Cohen’s d* |
| ROC AUC | 1 (0) | 9.7** | 3.67 | 1 (0) | 9.91** | 3.74 |
| Acc. | 1 (0.01) | 61.95** | 23.41 | 1 (0) | 17.99** | 6.80 |
| F1 score | .96 (0.09) | 38.45** | 14.53 | 1 (0) | 25.25** | 9.54 |
| Acc. = Balanced accuracy; * *p* < .05; ** = *p* < .001 | | | | | | |

The results of the between-participants classification analyses are presented in Table ST2. mean ROC AUCs, mean balanced accuracies and mean F1 scores were higher than baseline values, all *p_s_* < .001, all *Cohen’s d_s_* > 2.9. Frequentist ANOVAs suggested that there was no effect of Group on these measures, all *F*_s_(2, 42) < 3.18, all *p*_s_ > .052, but it was not supported by the *BF*_10_ (*BF*_10_ range: 0.51–0.95), unregistered analyses.

Table ST2: Results of the between-participants classification analyses for the two new countermeasure groups in Experiment 2

|  | Enhanced countermeasure | | | Feedback | | |
| --- | --- | --- | --- | --- | --- | --- |
|  | Mean (±*SD*s) | *t*(14) | *Cohen’s d* | Mean (±*SD*s) | *t*(14) | *Cohen’s d* |
| ROC AUC | .99 (0.03) | 7.66** | 2.9 | 1 (0) | 7.57** | 2.86 |
| Acc. | .94 (0.05) | 12.5** | 4.73 | 1 (0.02) | 14.44** | 5.46 |
| F1 score | .92 (0.06) | 11.44** | 4.32 | .99 (0.02) | 12.03** | 4.55 |
| Acc. = Balanced accuracy; * *p* < .05; ** = *p* < .001 | | | | | | |
